# In Situ Incorporation of Atomically Precise Au Nanoclusters within Zeolites for Ambient Temperature CO Oxidation

**DOI:** 10.3390/nano13243120

**Published:** 2023-12-12

**Authors:** Siriluck Tesana, John V. Kennedy, Alex C. K. Yip, Vladimir B. Golovko

**Affiliations:** 1 School of Physical and Chemical Sciences, University of Canterbury, Christchurch 8041, New Zealand; s.tesana@gns.cri.nz; 2 The MacDiarmid Institute for Advanced Materials and Nanotechnology, Wellington 6140, New Zealand; j.kennedy@gns.cri.nz; 3 National Isotope Centre, GNS Science, Lower Hutt 5010, New Zealand; 4 Department of Chemical and Process Engineering, University of Canterbury, Christchurch 8041, New Zealand

**Keywords:** gold nanoclusters, in situ encapsulation, LTA zeolite, CO oxidation

## Abstract

Preserving ultrasmall sizes of metal particles is a key challenge in the study of heterogeneous metal-based catalysis. Confining the ultrasmall metal clusters in a well-defined crystalline porous zeolite has emerged as a promising approach to stabilize these metal species. Successful encapsulation can be achieved by the addition of ligated metal complexes to zeolite synthesis gel before hydrothermal synthesis. However, controlling the metal particle size during post-reduction treatment remains a major challenge in this approach. Herein, an in situ incorporation strategy of pre-made atomically precise gold clusters within Na-LTA zeolite was established for the first time. With the assistance of mercaptosilane ligands, the gold clusters were successfully incorporated within the Na-LTA without premature precipitation and metal aggregation during the synthesis. We have demonstrated that the confinement of gold clusters within the zeolite framework offers high stability against sintering, leading to superior CO oxidation catalytic performance (up to 12 h at 30 °C, with a space velocity of 3000 mL g^−1^ h^−1^).

## 1. Introduction 

The catalytic behavior of metal-based catalysts is strongly dependent on their particle size. This is especially true for metal nanoclusters (MNCs), whose unique properties are highly sensitive to the number of metal atoms in the core. The addition or subtraction of a single metal atom to the ultrasmall clusters can critically influence the catalytic activity [1,2,3]. Therefore, controlling and maintaining the sizes of MNCs is essential in the study of metal nanocluster-based catalysis. Capping ligands are generally used in the synthesis of metal colloids and MNCs to control the size and suppress the overgrowth of the metal particles. When such species are used to make catalysts, ligands can act as a barrier, prohibiting reactants from accessing active metal surfaces. Many strategies have been developed to preserve the ultrasmall sizes of metal particles, particularly during catalyst activation and catalytic reaction, where protecting ligands are removed [4]. Immobilization of the metal particles onto solid supports before removing the ligands is one common approach. However, depositing the metal particles onto the surface of the solid supports, in some cases, often cannot provide satisfactory stability to the metal particles [5,6,7]. Due to their high surface energy, naked ultrasmall metal particles tend to aggregate by Ostwald ripening or Smoluchowski ripening, hence, losing catalytically active surface [8]. Our team [9,10,11,12,13,14,15], as well as several other groups [8,16,17,18], have reported that controlling MNC aggregation can be both important and challenging.

In this respect, microporous aluminosilicate zeolites appear as a promising host for confining ultrasmall MNCs. A well-defined and rigid zeolite framework could offer better stability by accommodating MNCs inside their cavities, well-isolated from one another. In such cases, the ultrasmall MNCs would have a better chance to maintain their size and catalytically active surfaces. The great thermal stability of zeolites, their large specific surface area, and more importantly, the specific pore and cavity sizes could also facilitate unique reactant, product, and transition-state selectivity and suppress catalyst poisoning [19,20,21,22,23]. 

The challenging task is that the fabrication of such encapsulated MNCs within zeolite is not straightforward [24,25,26]. Common methods used for the preparation of zeolite-supported metal catalysts involve ion exchange and wet impregnation. In both cases, solvated metal complexes are introduced to the zeolite void space after the framework is formed. Therefore, the location and homogeneity of distribution of metal species and their sizes strongly depend on the diffusion of the metal precursor through the framework defined by the pore openings of a particular zeolite. It would be a major challenge to synthesize atomically precise metal particles using this approach. These post-synthetic protocols are generally restricted significantly for small-pore zeolites, where a majority of the metal species may not be able to access the micropores. Instead, they are likely to remain at the outer surface and sinter to form larger particles during the activation and reduction steps [27,28,29,30,31]. 

An alternative approach, an in situ encapsulation, where a ligated metal precursor is introduced to a zeolite synthesis gel before the framework formation may, therefore, be preferable [32,33,34,35,36,37,38,39,40,41,42,43,44,45,46,47,48,49]. Using this approach, zeolite building units can assemble around the metal species, occluding the metals within the resulting framework during crystallization. However, successful encapsulation can only be achieved if (1) the metal precursors possess adequate stability in a strongly alkaline media of zeolite synthesis, and (2) good interaction between the metal species and the zeolite building units is established. Therefore, choosing appropriate passivating ligands (e.g., amine-based [33,34,35,36,37,38,39,40,41,42,43] and mercaptosilane-based [44,45,46,47,48,49] ligands) that strongly coordinate with the metal precursors and promote the assembly of zeolite building units is a crucial step in this approach. Without the assistance of such ligands, the metal species tend to precipitate prematurely and severely agglomerate to form larger metal particles on the external surface of the zeolite or even bulk metal separated from zeolite hosts [33,34,35,36,44,45,46]. 

Bifunctional mercaptosilane-based ligands (such as 3-mercaptopropyl trimethoxysilane, MPTMS), among protecting ligands, have been reported to offer sufficient stability to simple metal precursors, such as H_2_PtCl_6_, Pd(NH_3_)_4_Cl_2_, H_2_IrCl_6_, (NH_4_)_3_RhCl_6_, AgNO_3_, and HAuCl_4_ [44,45]. Through a strong metal-S bond, the mercapto groups (-SH) of the ligands provide chemical protection to the metal precursors against reduction or hydrolysis, even in strong alkaline media. Meanwhile, the silane moieties of the ligand simultaneously induce condensation of the silicate oligomer around the metal precursors. The formation of covalent Si-O-Si or Si-O-Al bonds between the ligated metal precursors and the zeolite building units then encapsulates metal species. At the same time, the zeolite framework is being formed, allowing these simple metal species to be incorporated within the zeolite cavity without premature precipitation and/or severe aggregation.

This strategy, however, requires further reduction of these simple encapsulated ligated metal species using reductive thermal treatment to produce encapsulated metal nanoparticles (MNPs) or MNCs with clean surfaces. The conditions of such inevitable post-treatment have a prominent influence on the sizes and size distributions of the MNPs and MNCs [50]. The precise control of the metal particle size is challenging, especially for gold MNPs and MNCs, whose sizes are susceptible to the temperature and chemical atmosphere [51]. There exists a very narrow temperature range of reductive conditions that can achieve encapsulated, monodispersed Au MNCs with good control over the particle size [45].

**Figure 1:**
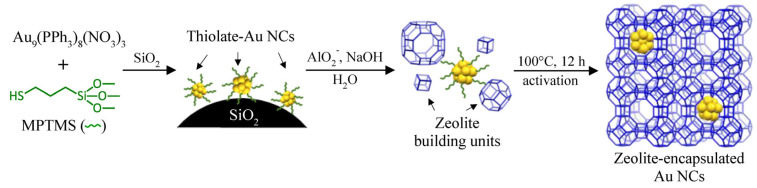
In situ encapsulation of atomically precise Au_9_ within Na-LTA zeolite.

In this study, we report the first successful attempt at in situ encapsulation of atomically precise gold nanoclusters (Au NCs) within the LTA zeolite void (Figure 1). 

Instead of gold complexes, pre-made ligated Au_9_ clusters, Au_9_(PPh_3_)_8_(NO_3_)_3_, were synthesized and used as precursors to the metal active sites. It was important to carry out a ligand exchange of the phosphine ligands with bifunctional (3-mercaptopropyl) trimethoxysilane (MPTMS) ligands prior to the hydrothermal synthesis of zeolite to ensure the compatibility and stability of the metal precursor in the synthesis media. It was hypothesized that the ultra-small Au_9_ clusters (~0.8 nm core size) with MPTMS ligands could match the LTA zeolite structure (~1.1 nm cavities with ~0.42 apertures). Indeed, we demonstrated that the zeolite-confined Au NCs maintained their ultra-small size and were resilient against sintering. Ambient temperature catalytic CO oxidation was used to investigate the catalytic reactivity and encapsulation efficiency of the LTA-encapsulated Au NCs, which were compared and contrasted with the post-impregnated Au NCs on the same zeolite.

## 2. Experimental Section

### 2.1. Materials Synthesis

#### 2.1.1. Synthesis of Au_9_(PPh_3_)_8_(NO_3_)_3_ Nanoclusters

Atomically precise Au_9_(PPh_3_)_8_(NO_3_)_3_ nanoclusters, denoted as ‘Au_9_’, were synthesized according to the protocol reported by Anderson et al. [14]. The ethanolic solution of NaBH_4_ (0.02 M, 90 mL) was added to the ethanolic solution of AuPPh_3_NO_3_ (0.048 M, 160 mL). The mixture was stirred at 1000 rpm for 2 h in the absence of light to obtain a deep red solution. Subsequently, insoluble impurities were filtered off, and the solvent was evaporated under reduced pressure. The obtained solid was redissolved in 20 mL of CH_2_Cl_2_ before filtration. The dark green precipitate yielded after further solvent removal. The product was then washed with THF and hexanes before being dissolved in methanol and subjected to crystallization via vapor diffusion of diethyl ether as an anti-solvent at 4 °C in the dark for 5 d. The dark green crystals were washed with diethyl ether and dried in vacuo. The yield of the Au_9_ was ca. 2.10 g, 60 ± 6 Au at%. 

Details of Au_9_ gold–phosphine cluster synthesis and characterization can be found in the Supporting Information, S3.1–S3.4.

#### 2.1.2. Ligand Exchange of Au_9_(PPh_3_)_8_(NO_3_)_3_ with (3-mercaptopropyl) Trimethoxysilane

The ligand exchange reaction of the Au_9_ with (3-mercaptopropyl) trimethoxysilane (MPTMS) was performed following the procedure reported by Woehrle et al., with minor modifications [52]. Typically, 20 equivalents of MPTMS (262 µL, 1.34 mmol) were added to a dark brown methanolic solution of Au_9_ (0.27 g, 0.067 mmol, 150 mL) while stirring. The mixture was stirred at 55 °C under an N_2_ atmosphere for 18 h. A trace amount of insoluble solids was separated by centrifugation. The crude product from the ligand exchange reaction was named ‘Au_9_-MPTMS’, and was characterized by NMR, MS, and UV-vis techniques (refer to the Supporting Information, S3.5) before using it in zeolite synthesis.

#### 2.1.3. Pre-Mixing of SiO_2_ with the Au_9_-MPTMS

For the synthesis of Na-LTA encapsulated Au NCs, fumed SiO_2_ (3.2 g, 0.053 mol) was suspended in a methanolic solution of the Au_9_-MPTMS ligand exchange product under agitation. After 1 h, the solvent was removed in vacuo. The obtained fumed SiO_2_ modified by Au_9_-MPTMS (MPTMS:SiO_2_ mol ratio of 1:40) was dried overnight under vacuum before being added to the aluminate solution for Na-LTA zeolite synthesis. 

For the synthesis of Na-FAU encapsulated Au NCs, colloid SiO_2_ LUDOX AM-30 (22.4 g, 30 wt% SiO_2_, 0.1 mol) was added to a methanolic solution of the Au_9_-MPTMS ligand exchange product under agitation. After the removal of methanol in vacuo, the colloidal SiO_2_ modified by Au_9_-MPTMS (MPTMS:SiO_2_ mol ratio of 1:60) was added to the aluminate solution for Na-FAU zeolite synthesis.

#### 2.1.4. Incorporation of Au_9_-MPTMS within Na-LTA Zeolite

The encapsulation of Au NCs within LTA zeolite was performed using a strategy similar to that reported by Wu et al. and Otto et al. [34,45]. Instead of simple ligated metal complexes, the fumed SiO_2_ modified by Au_9_-MPTMS was added to the aluminate solution before hydrothermal crystallization. Typically, NaOH (4.8 g, 0.12 mol) and NaAlO_2_ (6.0 g, 0.07 mol) were dissolved in 40 mL of Milli-Q water. While stirring (~750 rpm), the SiO_2_ modified by Au_9_-MPTMS and 22 mL of Milli-Q water were added to the mixture to form a homogeneous Au NCs-containing aluminosilicate gel with a molar ratio of 2.6 Na_2_O: 1.0 Al_2_O_3_: 1.5 SiO_2_: 93.6 H_2_O: 0.02 Au. The gel was aged at 60 °C for 4 h before being transferred into 60 mL Teflon-lined stainless steel autoclaves (ca. 40 g of gel in each) and crystallized at 100 °C for 16 h. The product was collected by centrifugation at 12,000 rpm for 5 min and washed with Milli-Q water. After being suspended in methanol overnight, the as-made ‘Au_9_-MPTMS@Na-LTA’ product was collected, dried overnight in vacuo, and further dried at 100 °C for 12 h in ambient air. The amount of Au clusters reported above (1.2 wt% Au loading, assuming 100% yield of Na-LTA) was adjusted to achieve 1.0 wt% Au loading in the final Au_9_-MPTMS@Na-LTA product (refer to the Supporting Information, S3.6).

For comparison, in the absence of MPTMS, an ‘Au_9_-PPh_3_@Na-LTA’ sample was prepared by adding a methanolic solution of Au_9_(PPh_3_)_8_(NO_3_)_3_ to the zeolite synthesis gel before the hydrothermal treatment. Through identical procedures to the Au_9_-MPTMS@Na-LTA sample, the aged gel of Au_9_-PPh_3_@Na-LTA was subjected to hydrothermal treatment at 100 °C for 16 h. The obtained product was collected, washed, and suspended in methanol in the same fashion. The as-made Au_9_-PPh_3_@Na-LTA was dried overnight in vacuo and further treated at 100 °C for 12 h in ambient air.

The yield of the as-made Au_9_-MPTMS@Na-LTA sample and Au_9_-PPh_3_@Na-LTA was ca. 9.42 g (96 ± 2% based on SiO_2_). The addition of Au_9_-MPTMS and Au_9_(PPh_3_)_8_(NO_3_)_3_ did not significantly affect the yield of the final products.

#### 2.1.5. Incorporation of Au_9_-MPTMS within Na-FAU Zeolite

The FAU-encapsulated Au NCs were prepared following the methods reported by Chen et al. and Otto et al., with minor modifications [39,45]. In the typical synthesis, NaAlO_2_ (1.82 g, 0.02 mol) and NaOH (6.24 g, 0.16 mol) were dissolved in 60 mL of Milli-Q water. The colloidal SiO_2_ modified by Au_9_-MPTMS was then added to the alumina solution while stirring (~750 rpm) to obtain the synthesis gel with the composition of 8.0 Na_2_O: 1.0 Al_2_O_3_: 10.1 SiO_2_: 376.5 H_2_O: 0.07 Au. The mixture was stirred (~750 rpm) at room temperature for 24 h. The synthesis gel was transferred into 60 mL autoclaves (*ca*. 40 g of gel in each) and crystallized at 100 °C for 15 h. The ‘Au_9_-MPTMS@Na-FAU’ product was collected, washed, and dried in the same manner as in the case of Au_9_-MPTMS@Na-LTA. 

The yield of the as-made Au_9_-MPTMS@Na-FAU sample was ca. 4.30 g (65 ± 4%, based on Al_2_O_3_). Of note, the addition of Au_9_-MPTMS did not significantly affect the yield of the FAU-based catalyst. Similar to Au_9_-MPTMS@Na-LTA, the amount of Au NCs added was adjusted to achieve Au_9_-MPTMS@Na-FAU with the targeted loading of 1.0 wt% Au (refer to the Supporting Information, S3.7).

### 2.2. Catalyst Activation

To remove the protecting ligands surrounding the Au NCs cores, the zeolite-encapsulated Au NCs were treated under ozone flow. The samples were exposed to O_3_ (170 μg mL^−1^ of the target concentration) for 1 h at different temperatures under magnetic stirring (500 rpm). This was achieved using a Schlenk flask placed in an aluminum block on a hotplate stirrer. The ozone was produced by ozone generator OL100H1DS, Yanco Industries, Ltd., with an initial O_2_ flow of 150 mL min^−1^.

### 2.3. Ambient Temperature CO Oxidation for Testing of Activity and Encapsulation Efficiency

Catalytic CO oxidation was performed in a stainless steel continuous-flow fixed-bed reactor. The reactor was operated in differential mode between 30 and 200 °C at atmospheric pressure with a gas hourly space velocity (GHSV) ranging from 3000 to 30,000 mL g^−1^ h^−1^. In a typical reaction, a gas mixture containing 1.0 vol% of CO, 10.5 vol% of O_2_, balanced with Ar and N_2_, was introduced into the reactor. Either 40 or 200 mg of the catalyst was loaded into the stainless steel reactor. The reactor was placed in a furnace controlled by a programmable temperature controller. The product gas was analyzed by an online GC (SRI Multiple Gas Analyzer, details in the Supporting Information, S4). All experiments were performed in duplicate.

## 3. Results and Discussion

### 3.1. Synthesis and Characterizations of LTA Zeolite-Encapsulated Au_9_ Nanoclusters

**Figure 2:**
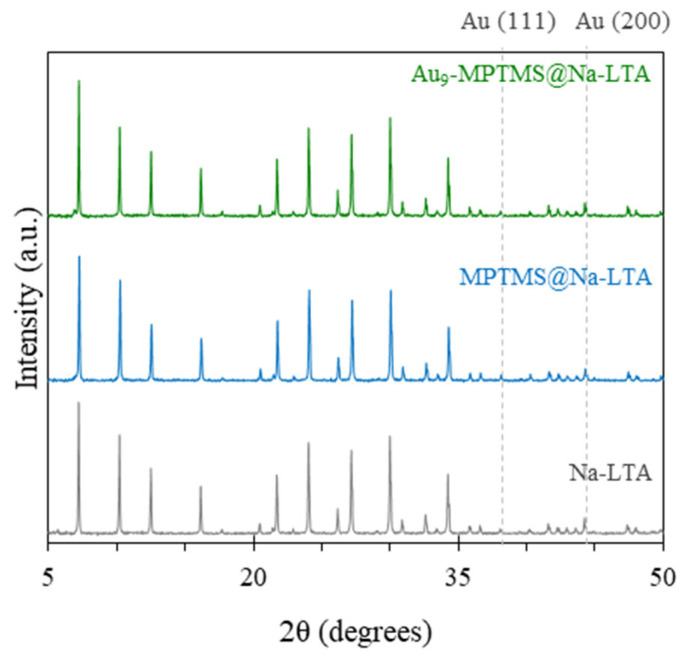
PXRD of Na-LTA zeolite-based samples synthesized in the presence of Au_9_-MPTMS and MPTMS, compared to Na-LTA.

Na-LTA zeolite was hydrothermally synthesized in the presence of the crude ligand exchange product, Au_9_-MPTMS. Without further addition of organic structure-directing agents, the LTA zeolite framework could be formed under relatively mild conditions (100 °C, 16 h). Structural evidence of the LTA zeolite phase and its purity were verified by PXRD analysis. As shown in Figure 2, PXRD of Au_9_-MPTMS@Na-LTA showed a characteristic peak pattern corresponding to the crystalline LTA zeolite, with approximately the same peak intensity as that of gold-free Na-LTA samples with and without the addition of MPTMS [53]. This result indicated that ligated Au_9_ nanoclusters, Au_9_-MPTMS, did not interfere with the hydrothermal crystallization process of the Na-LTA zeolite. The negligible difference in the PXRD peaks’ intensity between the samples suggested that a similar zeolite crystallinity level was systematically achieved.

The total Au content in all samples was measured using MP-AES elemental analysis. By adding SiO_2_-deposited Au_9_-MPTMS to the aluminate solution before hydrothermal crystallization, ca. 93 wt% of the introduced Au NCs were incorporated within the Na-LTA zeolite. The Au_9_-MPTMS@Na-LTA with 1.11 ± 0.02 wt% Au was achieved with excellent reproducibility, as confirmed by eight independent syntheses (Figure S3).

Without the MPTMS ligand exchange step, introducing Au_9_(PPh_3_)_8_(NO_3_)_3_ to the zeolite synthesis gel led to a very low incorporation efficiency. Only ca. 10 wt% of the Au precursors were found in the final Au_9_-PPh_3_@Na-LTA sample (ca. 0.12 wt% Au). These results highlight a significant contribution of the MPTMS ligand in promoting Au NC incorporation into Na-LTA zeolite. As reported by Iglesia et al., the alkoxysilane moiety of MPTMS can be hydrolyzed in alkaline media to form covalent Si-O-Si or Si-O-Al bonds with the zeolite building units, which then enforce the encapsulation of the metal species during the growth of zeolite crystal [44,45,46].

It is worth mentioning that such contributions of MPTMS were observed only when the ligand exchange between the Au NCs and MPTMS was conducted beforehand. A direct co-addition of the Au_9_ and MPTMS to the zeolite synthesis gel resulted in very low gold content (<0.2 wt%) in the final product, which is similar to the case of synthesis using only Au_9_ (i.e., in the absence of MPTMS). This is in contrast to the case of zeolite containing Au NPs, as reported by Iglesia and co-workers, where Au NPs can be encapsulated within zeolite voids via the direct co-addition of HAuCl_4_ and MPTMS to the similar zeolite synthesis gel, without a dedicated ligand exchange step beforehand [45]. This could be attributed to the properties of HAuCl_4_, which are more soluble, stable, mobile, and active compared to phosphine-protected Au_9_ NCs. The bonding between the Au^3+^ species of HAuCl_4_ and the mercapto group of MPTMS can be quickly established after the direct addition. In contrast, the ligand exchange from phosphine to MPTMS in Au NCs can only occur under suitable conditions (methanol as a solvent, 55 °C temperatures, and prolonged periods of time—18 h). Thus, a dedicated ligand exchange step is necessary for the encapsulation of the Au NCs within the zeolite.

Determining the size of the incorporated Au species was one of the biggest challenges in this study, especially since they were just a few Au atoms in size and were incorporated within the zeolite framework [32]. The MNP sizes with larger diameters (e.g., >2 nm) can be determined using high-resolution TEM. For example, ~2 nm-encapsulated MNPs (Pt, Pd, Ru, and Rh) within GIS zeolite (8-MR) are marginally visible in HR-TEM images [33]. While the smaller MNPs (~1.0–1.5 nm) in ANA (8-MR) and SOD (6-MR) are not visible using conventional TEM but can be observed using the high-angle annular dark-field high-resolution scanning transmission electron microscopy (HAADF HR-STEM) [33]. With HAADF HR-STEM, the Pt NCs with a diameter of 0.2–0.7 nm or even individual metal atoms are distinguishable (as bright spots) within the MCM-22 zeolite crystallites [54]. Unfortunately, the size of Au NCs could not be directly verified by electron microscopy, as they fall below the detection limit of the conventional TEM used in this study. Apart from the ultrasmall size of Au species, the thickness of the zeolite crystallites (~200–700 nm, Figure S4a,b) was another factor that made size determination via electron microscopy impossible. However, the absence of large Au particles (>2 nm) in the as-made Au_9_-MPTMS@Na-LTA sample containing 1.11 ± 0.02 wt% Au could be confirmed; Figure S4c,d.

In this work, the existence of Au NCs with a diameter smaller than 2 nm in the as-made Au_9_-MPTMS@Na-LTA sample was indirectly evidenced by MP-AES analysis, along with PXRD and UV-vis DRS. The absence of bulk Au peaks at 2*θ* of 38.1 and 44.3 in the PXRD of Au_9_-MPTMS@Na-LTA (Figure 2) suggested that the Au NCs retained their size and did not sinter to form larger Au crystallites (>10 nm) during the hydrothermal synthesis. Of note, the detection limits of PXRD are generally in the wide range of 1–10% by mass. However, these values vary significantly with the instrument and type of sample [55]. 

**Figure 3:**
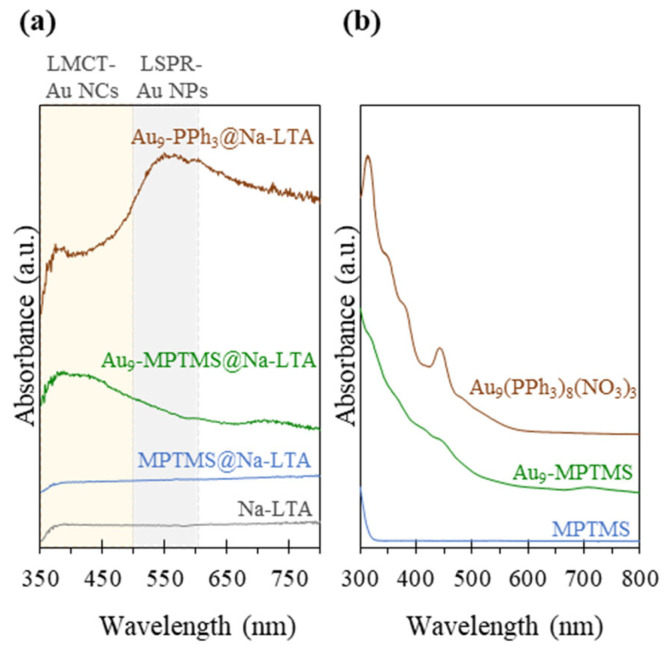
(**a**) UV-vis DRS of Na-LTA zeolite-based samples synthesized in the presence of Au_9_-PPh_3_, Au_9_-MPTMS, and MPTMS, compared to Na-LTA. (**b**) UV-vis spectra of methanolic solutions of Au_9_(PPh_3_)_8_(NO_3_)_3_, Au_9_-MPTMS, and MPTMS.

Apart from TEM and PXRD, UV-vis DRS was employed to monitor the formation of undesirably large Au NPs (>2 nm) due to cluster agglomeration. The size of the Au NPs could be roughly estimated from the position of the localized surface plasmonic resonance band (LSPR) in the UV-vis DRS. It is worth mentioning that such an estimation was not feasible for small Au NCs. Their optical properties are dominated by molecular-like single-electron transitions between quantized energy levels. As shown in Figure 3a, the Au_9_-MPTMS@Na-LTA sample did not exhibit detectable LSPR-Au NPs absorption bands (LSPR, at 500–600 nm) [56,57]. or sharp ligand-to-metal charge transfer peaks (LMCT, at 350–500 nm) [58] in UV-vis DRS. These data suggest that Au NPs larger than 2 nm were not present in the sample. While the existing ligand-exchanged Au_9_-MPTMS adducts did not exhibit LMCT peaks, the broad absorption feature at 350–500 nm matched well with the absorption feature of Au_9_-MPTMS in methanol, as shown in Figure 3b. Based on the UV-vis DRS results, MPTMS-stabilized Au NCs were found to have sufficient stability against sintering at the pH and temperature conditions of the LTA hydrothermal synthesis, similar to the case of Au^3+^ complexes reported previously [45]. 

Comparing UV-vis DRS of Au_9_-MPTMS@Na-LTA with that of Au_9_-PPh_3_@Na-LTA, a prominent LSPR-Au NP band was found in the latter case, synthesized in the absence of MPTMS. These results emphasize the contribution of the mercapto group of the MPTMS ligand in offering high stability to the Au species through a strong Au-S bond [59], suppressing Au NCs from severe aggregation and premature precipitation under strong alkaline crystallization conditions of the zeolite. Of note, the results also highlight the high sensitivity of the UV-vis DRS method in detecting plasmonic particles, even with a significantly lower Au content (0.12 wt%) in the Au_9_-PPh_3_@Na-LTA sample.

Using HAADF HR-STEM studies via an aberration-corrected microscope, the position of embedded MNPs/MNCs in the internal space of zeolite crystallites has been directly confirmed [31,32,54]. In this work, information about the location of metal species was indirectly deduced from the catalytic behavior of zeolite-incorporated metal. The catalytic activity of zeolite-encapsulated metal species and their higher stability have been widely used as evidence of successful encapsulation [33,34,35,44,45,46]. The results using this approach are discussed in depth in the following section.

LTA zeolite-incorporated Au_9_ nanoclusters were successfully synthesized via the in situ incorporation approach when the Au NCs were protected by MPTMS ligands. The product was obtained in significant yield with high zeolite crystallinity comparable to that of commercial zeolite. The sample showed great consistency in phase crystallinity and purity, the degree of incorporated Au, and absorption features across eight separately synthesized batches (Figure S3). More importantly, the sample exhibited high Au NC resilience against metal agglomeration and precipitation during the hydrothermal synthesis of zeolite.

### 3.2. Catalysts Activation

Since the pre-made Au clusters were employed for the synthesis of Au_9_-MPTMS@Na-LTA catalysts, H_2_ reduction treatment used to form Au NCs inside zeolite via the direct co-addition of HAuCl_4_ and MPTMS to the similar zeolite synthesis gel was not required. However, post-synthesis treatment was necessary to remove ligands and open the metal active sites for the catalytic CO oxidation (Figure 1). Calcination (at 400 to 600 °C) has been reported to offer complete ligand removal for thiol-capped Au NCs and Au NPs [60,61,62]. In some cases, the full removal of protecting ligands did not result in greater catalytic performance but led to the inactivity of the catalyst [62]. Conversely, remarkable CO oxidation performance without the complete removal of ligands was reported in some catalytic systems (e.g., CeO_2_-supported Au_25_(SR)_18_ [63] and Au_38_(SR)_24_ [64]). Therefore, the degree of ligand removal required to achieve high CO activity and good stability depends on the catalytic system.

The key challenge for the activation of Au_9_-MPTMS@Na-LTA catalysts was to remove the MPTMS ligands of the confined Au NCs while suppressing the severe agglomeration of Au NCs. For noble metals, such as Pt, Pd, and Ir, the removal of MPTMS ligands could be achieved by H_2_ treatment at 400 °C for 2 h [34]. Due to the weaker Au-S bonds, compared to the M-S bonds of the abovementioned noble metals (Au–S: 126 kJ mol^−1^; Pt–S: 233 kJ mol^−1^; Pd–S: 183 kJ mol^−1^; Ir–S: 206 kJ mol^−1^), it was expected that Au-bonded MPTMS can be removed under milder conditions [65,66]. Iglesia et al. reported the removal of MPTMS ligands from the Au^0^ NP surface via treatment at 400 °C in air followed by treatment at 300 °C under H_2_ [45]. However, under such conditions, we found that Au NCs sintered to form undesired larger Au NPs.

Thermogravimetric analysis (TGA) of pure Au_9_(PPh_3_)_8_(NO_3_)_3_ clusters showed an onset temperature for MPTMS ligand removal of ~230 °C (under N_2_ flow); Figure S5. However, there was no distinct difference in the TGA curves of Au_9_-MPTMS@Na-LTA compared to those of pure Na-LTA or MPTMS@Na-LTA, according to the low sensitivity limit of TGA. Thus, the decomposition temperature of individual species in Au_9_-MPTMS@Na-LTA cannot be identified. Moreover, the possibility of using temperature-programmed oxidation (TPO) to quantify the presence of ligands in the samples was investigated. Unfortunately, the ligand oxidation could not be quantified using the TPO experiment due to the CO_2_ generated from oxidation interfering with the thermal conductivity detector (TCD) signals with O_2_. 

To establish the activation conditions of Au_9_-MPTMS@Na-LTA, the sample was heated at temperatures varying from 200 to 400 °C for 1 h under static air (N.B.: b.p. of MPTMS is ~214 °C). The agglomeration of Au NCs after the calcination was monitored using UV-vis DRS. Activation conditions of Au_9_-MPTMS@Na-LTA were established at an early stage of the study using the sample with 0.75 ± 0.01 wt% Au, which was prepared in the same fashion as discussed above. The optimal activation conditions were later applied to the most promising sample, Au_9_-MPTMS@Na-LTA, with 1.11 ± 0.02 wt% Au in the following studies.

**Figure 4:**
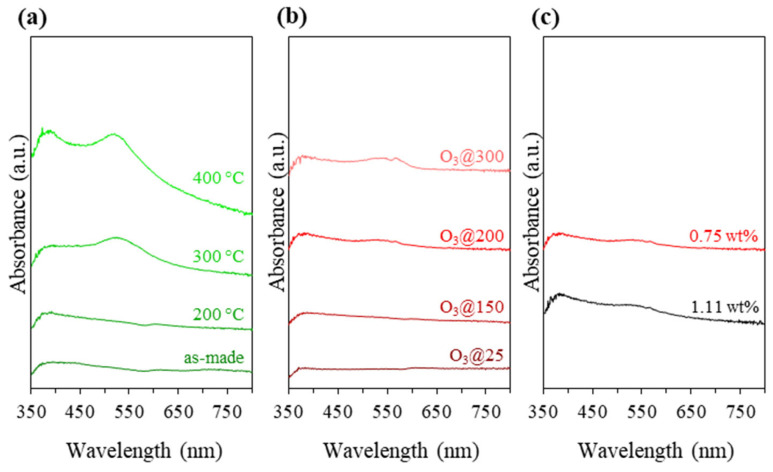
UV-vis DRS of Au_9_-MPTMS@Na-LTA (0.75 ± 0.01 wt% Au) (**a**) before and after calcination under static air at different temperatures of 200, 300, and 400 °C and (**b**) after ozonolysis at different temperatures of 25, 150, 200, and 300 °C. (**c**) UV-vis DRS of Au_9_-MPTMS@Na-LTA with 0.75 ± 0.01 and 1.11 ± 0.02 wt% Au after ozonolysis at 200 °C.

As shown in Figure 4a, the Au plasmonic band started to appear after calcination at 300 °C, indicating the onset of sintering of the Au species. Some ligand removal occurred during the heat treatment at 300 °C as well as undesirable Au agglomeration. There was no distinct difference between the UV-vis DRS of the 200 °C-treated sample and the untreated one, suggesting that severe Au sintering did not occur.

In the case of encapsulated noble MNP samples, such as Pt, Pd, Ru, Rh, and Ag, the degree of ligand removal could be inferred from the difference in metal surface areas derived from two approaches: (1) the mean diameter of metal particles obtained from electron micrographs and (2) H_2_ or O_2_ chemisorption by assuming spherical particles [33,34]. In the case of Au, the chemisorption of H_2_ and O_2_ is not feasible due to their high dissociation activation barriers [67]. Instead, CO chemisorption was used to obtain the surface area and derive the mean diameter of Au NPs within the zeolite framework [45]. However, the mean diameter of such Au NPs was not proportional to the total CO uptake by Au NPs encapsulated in zeolite. This is because the total CO uptake resulted from chemisorption on the Au species and the zeolite framework [68]. Therefore, FTIR spectra of the adsorbed CO must be recorded along with CO chemisorption to distinguish between CO adsorbed on Au NPs and zeolite, leading to a more complex monitoring approach.

In this work, the successful removal of the stabilizing ligands was inferred from the catalytic CO oxidation performance of the activated catalysts. The hypothesis was that if there was sufficient ligand removal, it would result in opened active sites, and the Au catalyst would show high CO oxidation activity at a relatively low reaction temperature. This hypothesis is based on earlier reports that ultra-small Au particles are active at much lower temperatures than larger Au species [69,70,71,72,73,74,75]. 

The CO oxidation was carried out in a fixed-bed flow reactor under atmospheric pressure at various temperatures, varying from 50 to 200 °C. As expected, without ligand removal, the Au_9_-MPTMS@Na-LTA sample was inactive for CO oxidation in the temperature range studied. Surprisingly, after the conventional calcination under static air, all samples remained inactive. Insufficient ligand removal was possibly the main reason for the inactivity of the sample calcined at 200 °C, whereas the formation of larger agglomerated Au NPs was likely responsible for the inactivity of the samples treated at 300 and 400 °C. The inactivity in low-temperature CO oxidation due to the formation of large Au NPs generally agreed with the previous reports in the literature [69,70,71,72]. 

Since the removal of protecting ligands and the suppression of Au NC agglomeration could not be achieved simultaneously using conventional heat treatment, ozonolysis as a chemical treatment under milder conditions was explored. Ozone is the highly reactive oxidizing agent that allows oxidative removal of organic moieties under lower temperatures, reducing the chance of cluster agglomeration [13,76,77]. Adopting the ozonolysis approach, the Au_9_-MPTMS@Na-LTA sample was treated under ozone flow at 25, 150, 200, and 300 °C for 1 h. A small change in the UV-vis DR spectra of all samples after ozone treatment is shown in Figure 4b. An O_3_@300 sample featured the most pronounced plasmonic band, while the rest of the O_3_-treated samples showed a negligible change in absorption intensity at the LSPR-Au NP absorption region of 500–600 nm. 

The catalytic CO oxidation testing using O_3_-treated samples was performed in the same manner as conventionally calcined samples. All O_3_-treated samples showed their catalytic CO activity at a reaction temperature of 200 °C, except O_3_@25, which was found inactive in the entire reaction temperature range (Figure S6). O_3_@150 showed poor performance, especially at a low reaction temperature (≤100 °C). Even though UV-vis DRS and TEM of the post-reaction samples of O_3_@25 and O_3_@150 (Figure S7) showed a very low degree of Au agglomeration, these ozonolysis conditions did not allow for adequate ligand removal for low-temperature CO oxidation to take place. O_3_@200 and O_3_@300 showed a much better catalytic performance at low temperatures. O_3_@200 gave more than 44% conversion and yield at 50 °C, and it was almost 50% higher than that of O_3_@300 (Figure S6). The greater population of the larger LSPR-Au NPs in the O_3_@300 sample (Figure 4b) was likely responsible for its lower activity.

According to the UV-vis DRS and TEM of the samples recovered after a catalytic test (Figure S7), the formation of larger LSPR-Au NPs during the catalytic test could be clearly confirmed in both O_3_@200 and O_3_@300 samples. As expected, the O_3_@300 post-reaction sample showed a greater degree of cluster aggregation due to a higher MPTMS-removal efficiency during ozonolysis. The majority of sintered Au NPs in the post-reaction samples were in the range of 2–5 nm, while a minority >10 nm in diameter was found only in the O_3_@300 post-reaction sample.

Ozonolysis combined with thermal treatment at 200 °C was shown to be the most promising activation approach for the Au_9_-MPTMS@Na-LTA as it resulted in adequate ligand removal, as indicated by the best catalytic activity at ~50 °C. In addition, these results also indicated a relatively high ratio of surviving Au NCs to sintered Au NPs in the O_3_@200 post-reaction sample. Ozonolysis at 200 °C was, therefore, applied to the most promising sample, Au_9_-MPTMS@Na-LTA, with 1.11 ± 0.02 wt% Au. As shown in Figure 4c, there was no distinct difference in the UV-vis DRS of the sample compared to the one with 0.75 ± 0.01 wt% Au, suggesting that severe Au sintering did not occur in the sample with a higher Au content.

### 3.3. Catalytic CO Oxidation

The in situ incorporation approach aimed to encapsulate the Au NCs within the zeolite voids, enabling the greater stability of the clusters against sintering during catalytic reaction. Detailed catalytic CO oxidation studies discussed below confirm the efficiency of the Au NC encapsulation achieved using the in situ incorporation approach. 

#### 3.3.1. Effect of the Incorporation Approach—In Situ vs. Post-Incorporation

Since the catalytic performance of supported Au catalysts in low-temperature CO oxidation is known to strongly depend on the Au particle size [69,70,71,72,73,74,75] (as well as the support types [78,79]), sintering of the Au species was hypothesized to reduce the catalytic performance, as already discussed in Section 3.2 Catalyst Activation. With the higher stabilization given by the incorporation inside of the zeolite framework, the confined Au NCs were proposed to maintain their ultra-small size and be resilient against sintering and, hence, show a high catalytic CO oxidation activity. At the same time, it was hypothesized that if Au NCs were located at the external surface, their sintering would be more pronounced due to poorer stabilization. Thus, we compared the catalytic activity of the Au NCs confined within the zeolite framework, prepared using in situ incorporation (denoted as inst-Au_9_-MPTMS@Na-LTA), and Au NCs immobilized on the external surface of the zeolite, prepared using post-impregnation (imp-Au_9_-MPTMS@Na-LTA; synthesis details are described in Supporting Information, S3.10).

**Figure 5:**
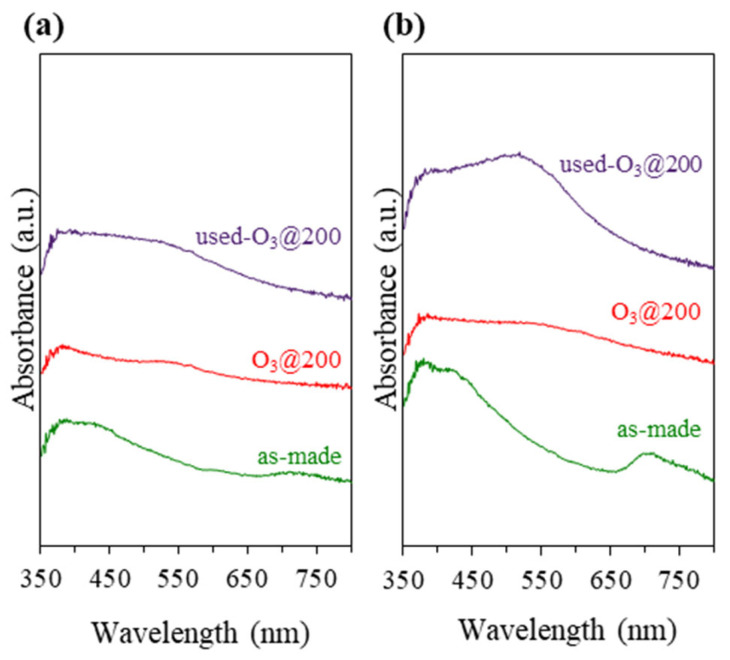
UV-vis DRS of as-made, O_3_@200-treated, and post-reaction samples of (**a**) inst-Au_9_-MPTMS@Na-LTA (1.11 ± 0.02 wt% Au) and (**b**) imp-Au_9_-MPTMS@Na-LTA (0.75 ± 0.03 wt% Au). Reaction conditions: T_max_ of 100 °C, a total reaction time of 12 h.

**Figure 6:**
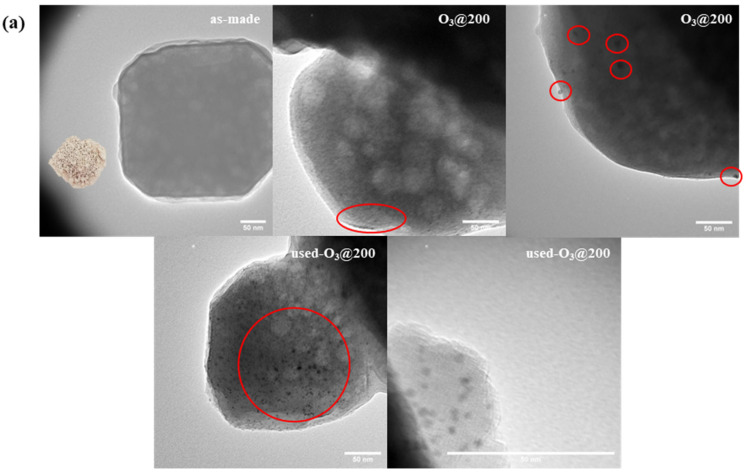

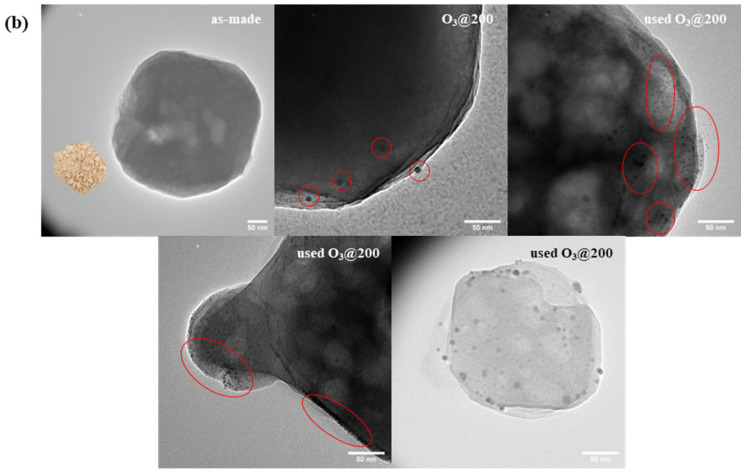
TEM images of as-made, O_3_@200-treated, and post-reaction samples of (**a**) inst-Au_9_-MPTMS@Na-LTA and (**b**) imp-Au_9_-MPTMS@Na-LTA. Reaction conditions: T_max_ of 100 °C, a total reaction time of 12 h. Au NPs are highlighted in red circles. Scale bars are 50 nm. Photographs of the as made samples are shown as inserts in the as-made TEM images.

With the initial Au loading of 1.2 wt%, Au_9_-MPTMS was introduced before and after the zeolite framework formation to give inst-Au_9_-MPTMS@Na-LTA and imp-Au_9_-MPTMS@Na-LTA, respectively. A much lower Au loading in the final product of imp-Au_9_-MPTMS@Na-LTA (0.75 ± 0.03 wt%) compared to that of inst-Au_9_-MPTMS@Na-LTA (1.11 ± 0.02 wt%) was confirmed by the MP-AES analysis. This result indicated that the in situ incorporation approach allowed Au NCs to incorporate with LTA zeolite better than in the case of post-impregnation. UV-vis DRS of as-made samples revealed much more apparent absorption features of Au_9_-MPTMS at ~440 and ~720 nm in the case of imp-Au_9_-MPTMS@Na-LTA in comparison to inst-Au_9_-MPTMS@Na-LTA (Figure 5, as-made). This result correlated with the more intense red–brown color of the imp-Au_9_-MPTMS@Na-LTA (Figure 6, as-made), implying that the majority of Au NCs were located on the external surface of Na-LTA. This is because the size of Au_9_-MPTMS (>0.8 nm) was larger than the pore opening of Na-LTA (0.42 nm). The introduction of the clusters to Na-LTA after the framework formation has not allowed Au_9_-MPTMS to access the zeolite pore.

**Figure 7:**
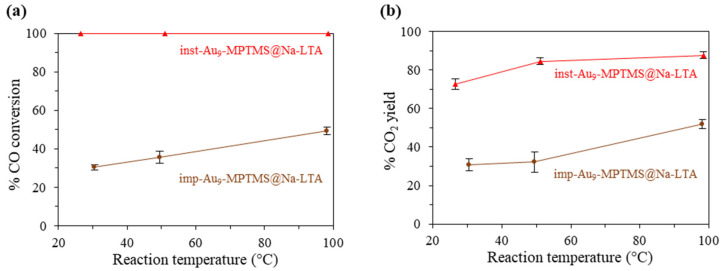
(**a**) % CO conversion and (**b**) % CO_2_ yield in CO oxidation catalyzed by O_3_@200-inst-Au_9_-MPTMS@Na-LTA (1.11 ± 0.02 wt% Au) and O_3_@200-imp-Au_9_-MPTMS@Na-LTA (0.75 ± 0.03 wt% Au). Reaction conditions: GHSV of 3000- or 2030-mL g^−1^ h^−1^, a total Au loading of 2.2 mg, 30–100 °C.

Before the catalytic test, the samples were ozonolyzed at 200 °C for 1 h. The formation of Au NPs during the activation cannot be confirmed by UV-vis DRS (Figure 5, O_3_@200). Nevertheless, TEM images (Figure 6, O_3_@200) revealed numerous dark spots corresponding to Au NPs, especially in the case of imp-Au_9_-MPTMS@Na-LTA. With the same total amount of gold per catalyst sample loaded into the reactor, the O_3_@200-inst-Au_9_-MPTMS@Na-LTA gave significantly higher CO conversion and CO_2_ yield compared to the O_3_@200-imp-Au_9_-MPTMS@Na-LTA at all reaction temperatures (Figure 7). Impressively, using the O_3_@200-inst-Au_9_-MPTMS@Na-LTA, 100% conversion was achieved at 30 °C, the lowest temperature used in this study, and remained the same at 50 and 100 °C. Moreover, the catalyst exhibited 100% conversion even after 12 h of catalytic testing, indicating excellent stability of this catalyst (Figure S15). A higher degree of Au agglomeration during the catalytic test in the case of the O_3_@200-imp-Au_9_-MPTMS@Na-LTA was likely responsible for its lower catalytic activity compared to that of O_3_@200-inst-Au_9_-MPTMS@Na-LTA. This was confirmed by UV-vis DRS and TEM images of the post-reaction samples (Figure 5 and Figure 6, used-O_3_@200), where (1) a more pronounced LSPR band of sintered Au NPs and (2) a higher population of Au NPs visible in TEM, on the edge of zeolite or even dislodged Au NPs, were found in the case of the O_3_@200-imp-Au_9_-MPTMS@Na-LTA.

All these results suggested that Au NCs confined within the zeolite framework by the in situ incorporation have better stability during CO oxidation than surface-bound Au NCs prepared by post-impregnation. The excellent stability of the Au NCs led to superior catalytic performance, achieving 100% CO conversion and 73–88% CO_2_ yield for up to 12 h time-on-stream. Hence, it could be concluded that the in situ incorporation approach offers Au NCs higher stability against agglomeration during the catalytic reaction in contrast to the post-impregnation approach.

#### 3.3.2. Effect of Zeolite Framework—LTA vs. FAU

As shown in Figure 7, CO_2_ yield was ~12–27% lower than CO conversion in the temperature range of 30–100 °C using the inst-Au_9_-MPTMS@Na-LTA catalyst. A greater difference in CO conversion cf. CO_2_ yield was found at lower temperatures (by 27% at 30 °C, and by 12% at 100 °C). The molecular adsorption of CO on zeolite counter ions and Au active sites in the zeolite-supported Au catalysts was previously reported as a reason for this carbon imbalance [45,68]. By conducting control experiments under the same conditions as the catalytic test but without O_2_–passing 1.0% CO in Ar through the samples at 30–100 °C, the amount of CO adsorbed could be determined. Without gold (O_3_@200-MPTMS@Na-LTA), a 4–7% loss of CO was found; in the presence of gold (O_3_@200-Au_9_-MPTMS@Na-LTA), a 10–14% loss was observed. Therefore, the carbon balance issue can be partially explained by the physisorption of CO within zeolite and the chemisorption of CO on Au [80]. Another possible explanation could be the adsorption of produced CO_2_ on the zeolite surface; however, further experiments are required to confirm this hypothesis.

Apart from the molecular adsorption of CO and CO_2_, the mass transfer rate of CO, O_2_, and CO_2_ within the LTA framework could also affect the catalytic reaction, especially when the conversion of CO to CO_2_ takes place at the Au NC active sites encapsulated within the zeolite framework. Since access to the cages, cavities, or channels of a zeolite is controlled by the largest free path, CO, O_2_, and CO_2_ with kinetic diameters of ~0.38, 0.35, and 0.33 nm, respectively, are allowed to enter the alpha cavity of Na-LTA (maximum aperture 0.42 nm). By using zeolite with a larger cavity aperture, the mass transfer could be generally improved. Nevertheless, the zeolite cavity diameter itself is considered a critical factor for successful in situ encapsulation. Thus, for the study of the mass transfer rate effect, a Na-FAU zeolite was chosen as it possesses the same maximum cavity diameter (of ~1.1 nm) as Na-LTA but has larger apertures of 0.74 nm [53,81].

Au_9_-MPTMS@Na-FAU was fabricated using the in situ incorporation approach, analogous to the Au_9_-MPTMS@Na-LTA (see Supporting Information, S3.7). Both Au_9_-MPTMS@Na-LTA and Au_9_-MPTMS@Na-FAU were treated under O_3_ at 200 °C before catalytic CO oxidation under the same conditions. 

**Figure 8:**
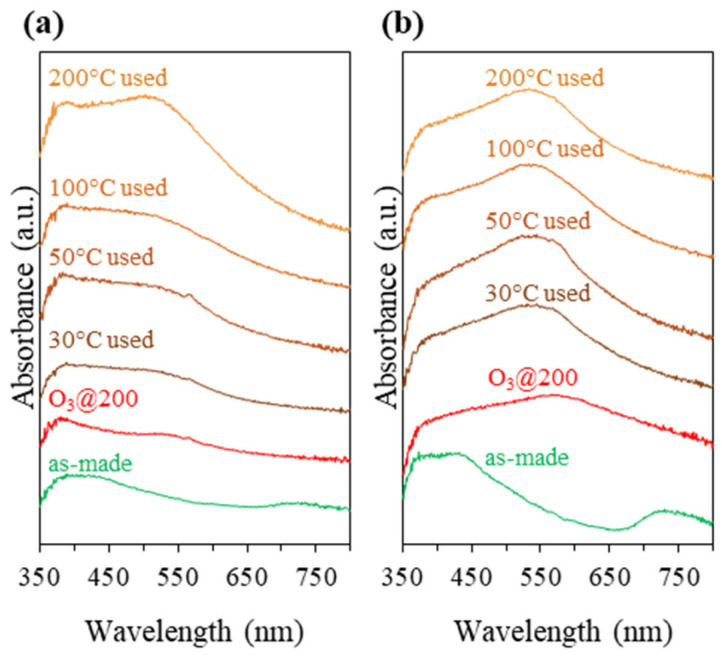
UV-vis DRS of as-made, O_3_@200-treated, and post-reaction samples of (**a**) Au_9_-MPTMS@Na-LTA (1.11 ± 0.02 wt% Au) and (**b**) Au_9_-MPTMS@Na-FAU (0.96 ± 0.05 wt% Au). Reaction conditions: temperatures of 30, 50, 100, and 200 °C; reaction time of 2 h.

Synthesis optimization experiments showed that to achieve the target Au content of 1.0 wt%, twice the amount of Au was introduced to the Na-FAU zeolite synthesis gel (~2.4 wt% Au) compared to that of Na-LTA (~1.2 wt% Au). Interestingly, the Au_9_-MPTMS@Na-FAU sample with 0.96 ± 0.05 wt% Au showed two prominent absorption bands at ~440 and ~740 nm (Figure 8b, as-made), which can be attributed to the Au_9_-MPTMS, similar to the case of imp-Au_9_-MPTMS@Na-LTA (Figure 5b, as-made). From the UV-vis DRS results, a higher density of Au NCs located at the outer surface of the FAU zeolite crystallite in comparison to that of LTA could be implied.

**Figure 9:**
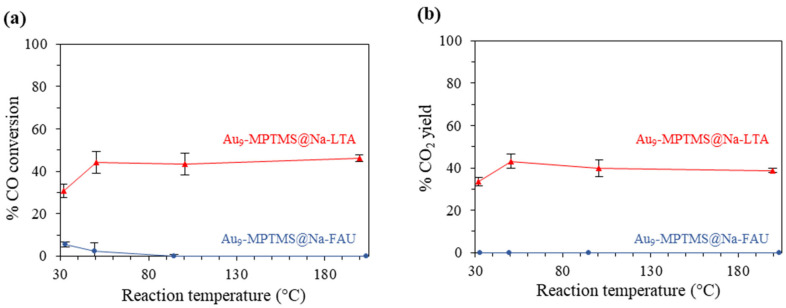
(**a**) % CO conversion and (**b**) % CO_2_ yield in CO oxidation catalyzed by O_3_@200-Au_9_-MPTMS@Na-LTA (1.11 ± 0.02 wt% Au) (raw data are shown in Figure S16) and O_3_@200-Au_9_-MPTMS@Na-FAU (0.96 ± 0.05 wt% Au). Reaction conditions: GHSV of ~30,000 mL g^−1^ h^−1^, catalyst loading of ~ 40 mg, total Au loading of 0.4 mg, 30–200 °C.

To compare the performance of the two catalysts, the gas hourly space velocity (GHSV) was adjusted to ~30,000 mL g^−1^ h^−1^ to ensure that the reaction was kinetically controlled. While the total Au loading for each catalytic test was maintained at 0.4 mg (~40 mg of the catalyst), each catalytic test was performed separately at 30, 50, 100, and 200 °C for 2 h. As shown in Figure 9, Au_9_-MPTMS@Na-LTA showed a lower conversion and yield due to the lower total Au loading and shorter contact time (higher GHSV). The sample gave 31 ± 3% CO conversion and 33 ± 2% CO_2_ yield at 30 °C. The conversion and yield increased with the increase in reaction temperature from 30 to 50 °C, where 44 ± 5% conversion and 43 ± 3% yield were observed. In comparison, a further increase in the reaction temperature from 50 to 100 and 200 °C did not significantly change CO oxidation activity. This consistency of the catalytic activity could be explained by the unchanged UV-vis DRS profiles of Au_9_-MPTMS@Na-LTA before and after the catalytic test at 30, 50, and 100 °C, as shown in Figure 8a. This result highlights that LTA could offer great sintering resistance to the Au species during the catalytic reaction. TEM, however, revealed some degree of Au sintering in the post-reaction samples of Au_9_-MPTMS@Na-LTA (used at 30, 50, and 100 °C). In comparison, the TEM of a post-reaction sample at 200 °C showed the highest numbers of sintered Au NPs among all used Au_9_-MPTMS@Na-LTA catalysts (Figure S8).

Surprisingly, the Au_9_-MPTMS@Na-FAU showed no catalytic activity in CO oxidation under the same conditions (Figure 9). The sample showed the highest CO loss (4–6%) at 30 °C, which was likely due to the adsorption rather than the conversion since no CO_2_ product could be detected up to 200 °C. The severe sintering of Au NCs to form larger Au NPs was likely responsible for the inactivity of the activated Au_9_-MPTMS@Na-FAU sample since the sample featured a broad plasmonic band even before the catalytic test (Figure 8b, O_3_@200). Such an intense plasmonic band after the O_3_ treatment was not found in any other samples reported in this work, not even in the imp-Au_9_-MPTMS@Na-LTA sample, which was prepared by the post-impregnation method. Thus, the more accessible FAU zeolite framework must, indeed, have a strong influence on this pronounced sintering phenomenon. 

It is possible that the larger pore opening of FAU not only allows CO and O_2_ to easily access the Au active sites but also allows Au NCs species to easily migrate out of the cavities and sinter with other Au particles, resulting in lower catalyst performance. The other hypothesis was that most Au species in the Au_9_-MPTMS@Na-FAU sample were not encapsulated within the FAU framework but were instead located on the external surface zeolite crystallites (which would account for the more pronounced features in Figure 8b, as made). Therefore, Au NCs in such samples showed low stability, undergoing severe agglomeration during catalyst activation and the catalytic test, as evidenced by the rather pronounced plasmonic band. The intensity of plasmonic bands in the post-catalytic samples of Au_9_-MPTMS@Na-FAU was even more significant than in the case of imp-Au_9_-MPTMS@Na-LTA, implying that sintering is facilitated by the chemical nature of the FAU sample (e.g., Si/Al ratio).

#### 3.3.3. Effect of Incorporated Au Species–Au NCs vs. Au NPs

Earlier, it was proposed that the formation of larger Au NPs due to the aggregation of Au NCs during the catalyst activation and/or the catalytic test was responsible for the lower catalytic activity or inactivity of the zeolite-supported Au catalysts. Under this hypothesis, the zeolite-supported ultra-small Au NCs were proposed to be the major active species responsible for CO oxidation at low temperatures, while it was hypothesized that the larger Au NPs were less active or inactive in CO oxidation under the same conditions. However, the size regime of the active Au species in this catalytic system cannot be verified due to the co-existence of Au NCs and Au NPs, as confirmed by the UV-vis DRS and TEM of all post-reaction samples. To directly compare the CO oxidation performance of the zeolite-supported Au catalyst with different sizes of incorporated Au species, AuNPs-MPTMS@Na-LTA was prepared following the procedure established by Iglesia et al. [45]. With a similar in situ incorporation strategy, the same Na-LTA zeolite host (Si/Al in the synthesis gel of ~0.8), and similar final Au content, the Au particle size was considered as the only different parameter influencing the catalytic performance of the two samples.

**Figure 10:**
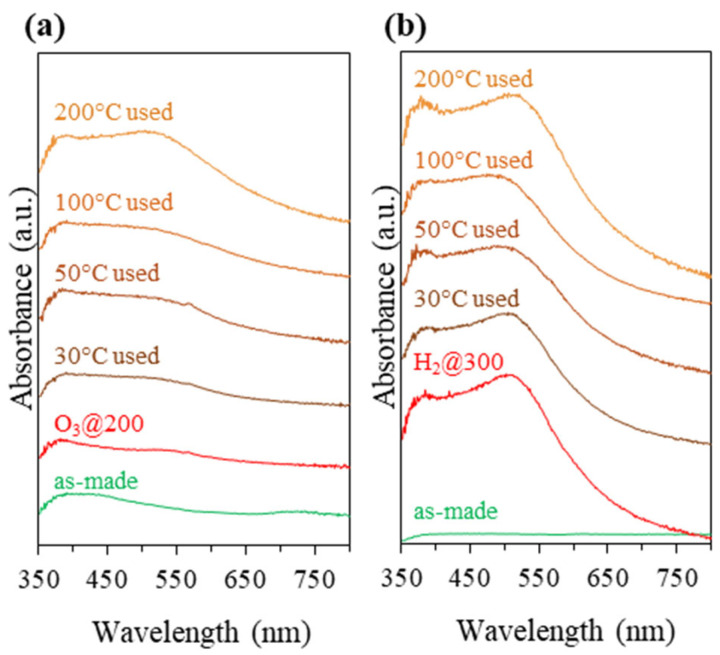
UV-vis DRS of as-made, activated, and post-reaction samples of (**a**) Au_9_-MPTMS@Na-LTA (1.11 ± 0.02 wt% Au) and (**b**) AuNPs-MPTMS@Na-LTA (1.16 ± 0.02 wt% Au). Reaction conditions: temperature of 30, 50, 100, and 200 °C; reaction time of 2 h.

The AuNPs-MPTMS@Na-LTA was obtained with a 98 ± 2% yield based on SiO_2_, comparable to the yield reported in the literature [45]. As confirmed by PXRD, AuNPs-MPTMS@Na-LTA showed a similar crystallinity to Au_9_-MPTMS@Na-LTA (Figure S11). A slightly higher Au content was found in the former case (1.16 ± 0.02 wt% Au cf. 1.11 ± 0.02 wt% Au) due to a higher amount of introduced gold (1.3 wt% Au cf. 1.2 wt% Au). Noteworthy, the formation of Au NPs in the AuNPs-MPTMS@Na-LTA sample did not take place until post-synthesis thermal treatment under air and later under H_2_, where MPTMS ligands were removed and, consequently, Au^3+^ was reduced to Au^0^. Consistent with the previous report, H_2_ reduction at 300 °C resulted in a sharp increase of the plasmonic band intensity with λ_max_ of 510 nm, indicating Au NPs (Figure 10b, H_2_@300) [45]. Based on the position of the LSPR band maximum, the size of the Au NPs could be estimated to be between 2 and 2.5 nm [82], or close to 279 Au core atoms (2.25 nm) [83]. 

Comparing the UV-vis DRS of the two activated samples with a similar Au loading (Figure 10, O_3_@200 cf. H_2_@300), O_3_@200-Au_9_-MPTMS@Na-LTA exhibited a broad band across the spectrum with a barely detectable plasmonic band. This may suggest that most Au species were in the cluster form, whereas H_2_@300-AuNPs-MPTMS@Na-LTA showed a prominent plasmonic peak, confirming the presence of larger Au NPs. However, further agglomeration of the Au species in both samples during the catalytic test was confirmed by a more intense and broader plasmonic band of the post-reaction samples. 

**Figure 11:**
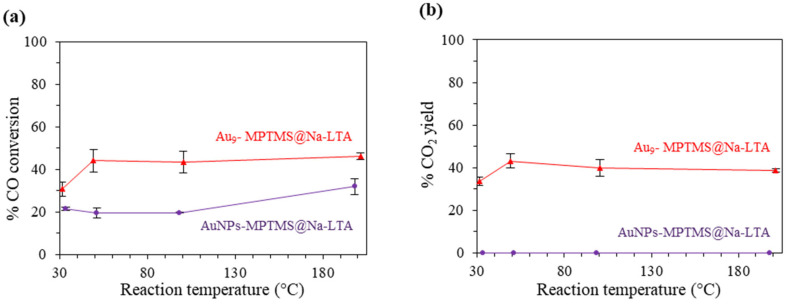
(**a**) % CO conversion and (**b**) % CO_2_ yield in CO oxidation catalyzed by O_3_@200-Au_9_-MPTMS@Na-LTA (1.11 ± 0.02 wt% Au) and H_2_@300-AuNPs-MPTMS@Na-LTA (1.16 ± 0.02 wt% Au). Reaction conditions: GHSV of ~30,000 mL g^−1^ h^−1^, catalyst loading of ~40 mg, total Au loading of 0.4 mg, 30–200 °C.

Catalytic testing was performed similarly to the test discussed earlier. Each catalytic test was performed separately at 30, 50, 100, and 200 °C, for 2 h, with a GHSV of 30,000 mL g^−1^ h^−1^. As shown in Figure 11, AuNPs-MPTMS@Na-LTA did not show any CO_2_ production up to 200 °C. A 20–30% CO loss found across 30–200 °C could possibly be attributed to the molecular adsorption of CO, as discussed in the earlier section. These results, therefore, supported the hypothesis that Au NCs, not Au NPs, acted as the active species in the low-temperature CO oxidation using zeolite-supported Au catalysts. 

A higher number of low-coordinated Au atoms in smaller supported Au particles was generally proposed as the origin of their excellent activity over larger Au particles due to their superior O_2_ binding or dissociation sites [69,70,71,72,84,85,86]. Moreover, the adsorption energies of both CO and O atoms on Au were reported to depend strongly on the coordination number of the Au atom to which they bind. Specifically, the theoretical calculations showed that both O and CO adsorption energies are lowered by up to 1 eV when the coordination number of Au atom is reduced from 9 in the case of Au (1 1 1) to 4 in the case of Au_10_ clusters [70]. However, the fact that the LTA-supported Au NPs with an average diameter of ~1.3 nm (as reported by Iglesia et al. [45]) or 2.0–2.5 nm (as estimated from the LSPR band position) did not show any CO oxidation at 30–200 °C remains inexplicable [87]. The effect of support types on the size thresholds of the active Au species might be one reason as it was shown earlier that the specific size regime of the most active Au species in low-temperature CO oxidation varied, depending on the type of oxide support [63,78,79].

## 4. Conclusions

In summary, we presented the fabrication of Au NC-based catalysts with clusters incorporated within the zeolite framework via the in situ incorporation of pre-made atomically precise Au_9_ clusters during hydrothermal synthesis of Na-LTA zeolite. We demonstrated the importance of the ligand exchange (phosphine to mercaptosilane ligands) for the highly reproducible successful synthesis of zeolite-incorporated gold clusters, with high stability of the Au_9_ clusters against sintering. The successful encapsulation of Au NCs was suggested by their superior catalytic CO oxidation performance. The catalyst illustrated good stability against sintering, maintaining a 100% CO conversion at 30 °C, up to 12 h, due to metal encapsulation. This result highlights the advantages of the restrictive framework of Na-LTA zeolite, which suppresses Au sintering, leading to the better performance of confined Au NCs in catalytic CO oxidation. The performance of this catalyst was superior to Au NCs immobilized on the external surface of LTA or encapsulated within the FAU framework with larger openings. Moreover, the unique catalytic activity of ultrasmall gold clusters compared to their larger gold nanoparticle counterparts was illustrated in this work. Ultra-small Au NCs have been shown to be major contributors to superior low-temperature catalytic CO oxidation performance. In contrast, even slightly larger Au NPs (2.0–2.5 nm) did not show any CO_2_ yield across the temperatures studied.

## Data Availability

The majority of data created during this study are available within this manuscript and its Supporting Information. All other data can be made available upon reasonable request to the corresponding authors.

## Supplementary Materials

The following supporting information can be downloaded at https://www.mdpi.com/article/10.3390/nano13243120/s1. The supporting information is available free of charge. It includes detailed information on material synthesis and characterizations (NMR, PXRD, mass spectra, UV-vis DRS, SEM, TEM, MP-AES results), as well as the setup for the catalytic CO oxidation test, including a process flow diagram, GC settings, typical chromatograms, and calibration plots (PDFs). (Reference [88] is cited in the supplementary materials).
