# Supplementary material for: In Situ Incorporation of Atomically Precise Au Nanoclusters within Zeolites for Ambient Temperature CO Oxidation
Source: Nanomaterials (Basel). 2023 Dec 12;13(24):3120. doi: 10.3390/nano13243120 (PMC10745642; doi:10.3390/nano13243120)
Supplement: Supplementary file 1 [file nanomaterials-13-03120-s001.zip › nanomaterials-2712374-supplementary.pdf]

Supporting Information

In Situ Incorporation of Atomically Precise Au Nanoclusters within Zeolites for Ambient Temperature CO Oxidation

Siriluck Tesana [1,2,3], John V. Kennedy [2,3], Alex C. K. Yip [2,4,*] and Vladimir B. Golovko [1,2,*]

1 School of Physical and Chemical Sciences, University of Canterbury, Christchurch 8041, New Zealand; s.tesana@gns.cri.nz
2 The MacDiarmid Institute for Advanced Materials and Nanotechnology, Wellington 6140, New Zealand; j.kennedy@gns.cri.nz
3 National Isotope Centre, GNS Science, Lower Hutt 5010, New Zealand
4 Department of Chemical and Process Engineering, University of Canterbury, Christchurch 8041, New Zealand
* Correspondence: alex.yip@canterbury.ac.nz (A.C.K.Y.); vladimir.golovko@canterbury.ac.nz (V.B.G.); Tel.: +64-3-3694086 (A.C.K.Y.); +64-3-3695942 (V.B.G.)

S1. Chemicals and gases

All reactants were analytical reagent grade and used without further purification. Chloroauric acid was prepared using 99.99 % pure gold, Regal Castings NZ. Hydrochloric acid (32 %) and nitric acid (70 %) were purchased from Ajax Finechem. Dichloromethane (99.9 %), sodium borohydride (99 %), diethyl ether (99 %), ethanol (99.9 %), methanol (99.99 %) and sodium hydroxide (97 %) were supplied by Fisher Scientific. Silver nitrate (99 %) was purchased from M&B laboratory chemicals. Triphenylphosphine (99 %), tetrahydrofuran (99.5 %), acetonitrile (99 %) and *n*-hexane (98 %), (3-mercaptopropyl) trimethoxysilane (MPTMS, 95 %), sodium aluminate (anhydrous, 50 – 56 % Al_2O_3 and 40 – 45 % Na_2O), fumed SiO_2 (anhydrous, 0.014 μm), colloidal SiO_2 Ludox AM-30 (30 % suspension in H_2O) and NMR solvents including CD_3OD, $CDCl_3$ and CD_2Cl_2 (99.8 %, over silver foil) were supplied by Merck. Gold standard solution (1000 ppm Au in 0.5 M HCl, N_2 flushed) was purchased from Acros Organics. Instrument grade H_2 (99.98 % with < 20 ppm H_2O), CO (99.999 %) were supplied by BOC. O_2 (99.2 % with < 10 ppm H_2O), CO_2 (Food grade) were supplied by Southern Gas Services Ltd. N_2 and Ar were instrument grade supplied by the in-house gas system.

S2. Characterization techniques

^{1}H and ^{31}P NMR spectra were collected at 298 K using a 400 MHz JOEL NMR. Mass spectrometry (MS) was performed using Bruker maXis 3G UHR-TOF MS with an electrospray ionization (ESI) source and positive-ion polarity. UV-Vis absorption spectra were recorded on a UV-Vis spectrophotometer (GBC Cintra 404) from a wavelength of 300 to 800 nm with a scan speed of 300 nm min^{-1}. Diffuse reflectance UV-visible spectra (UV-vis DRS) were recorded on the same spectrophotometer equipped with an integration sphere. The recording was performed in the wavelength range of 350 to 800 nm with a scan speed of 100 nm min^{-1} using a Spectralon® diffuse reflectance standard disk as reference. Powder X-ray diffraction (PXRD) of the $Au_9(PPh_3)_8(NO_3)_3$ nanoclusters was performed using the Agilent Technologies SuperNova X-ray diffractometer with Mo Kα radiation, while that of supported Au NCs was conducted using a Rigaku Smartlab diffractometer with Cu Kα radiation (40 kV and 30 mA) of wavelength 0.15 nm. Scanning electron micrographs (SEM) were taken with JEOL JSM 7000F field emission, high resolution scanning electron microscope at an accelerating voltage of 15 kV. Transmission electron micrographs were collected using Philips CM120 transmission electron microscope. Elemental analyzes were carried out by microwave plasma-atomic emission spectroscopy (MP-AES) using an Agilent 4210 MP-AES equipped with SPS 4 autosampler. Thermogravimetric analysis (TGA)

was performed using an Alphatech SDT Q600 TGA apparatus. Approximately 5 - 10 mg of samples were placed in an alumina crucible. The samples were heated from 25 to 800 ºC at a heating rate of 10 ºC min^{-1} under N_2 flow (100 mL min^{-1}).

S3. Materials synthesis and characterizations

S3.1. $HAuCl_4{\cdot}nH_2O$

Tetrachloroauric acid, $HAuCl_4{\cdot}nH_2O$ was prepared following the procedure reported by Glemser *et al.*[1] Pure gold metal was dissolved in the *aqua regia* solution, HNO_3: HCl at the ratio of 1:3 v/v. Excess acid was gently removed using a rotary evaporator to obtain the yellow-orange solid of $HAuCl_4$. The average molar percent yield of $HAuCl_4$ was 99 % by Au. Without further purification, $HAuCl_4$ was used to prepare $AuPPh_3Cl$, which was further converted to $AuPPh_3NO_3$, a precursor for $Au_9(PPh_3)_8(NO_3)_3$ clusters synthesis.

S3.2 $AuPPh_3Cl$

$AuPPh_3Cl$ was synthesized according to the procedure described by Anderson *et al.*[2] A 4.20 g (0.01 mol) of $HAuCl_4$ was dissolved in 40 mL of ethanol. The solution of 5.30 g (0.02 mol) triphenylphosphine in 150 mL was rapidly added into the $HAuCl_4$ solution. The mixture was stirred at 1000 rpm for 2 h until a white precipitate was obtained. The white solid was then collected by filtration through a sintered glass funnel porosity 3. The solid product was then washed with hot (*ca.* 50 °C) ethanol (3 x 20 mL). The remaining solid was dissolved in 60 mL of CH_2Cl_2. The filtrate was evaporated using a rotary evaporator to obtain the white solid. The crude product was dried in a vacuum desiccator overnight. CH_2Cl_2 (*ca.* 20 mL) was added carefully into the crude product to dissolve all the solid. The solution was transferred into small vials (2 x *ca.* 10 mL) which were placed into the big jar containing 50 mL of ethanol for vapour diffusion crystallization. The crystallization process was carried out at 4 °C for 2 d. The colorless needle and plate crystals were washed with a small portion of methanol and dried in a vacuum desiccator. The yield of the pure $AuPPh_3Cl$ product was *ca.* 4.62 g (9.4 mmol, *ca.* 95 % by Au). ^{31}P-NMR ($CDCl_3$ over silver foil): singlet, δ 33.2 ppm relative to 85 % H_3PO_4, which agrees with those reported in the literature.[2]

S3.3 $AuPPh_3NO_3$

$AuPPh_3NO_3$ was prepared according to the procedure detailed by Anderson *et al.*[2] with modification. A 3.97 g (0.008 mol) of $AuPPh_3Cl$ was dissolved in 50 mL of CH_2Cl_2. The solution of silver nitrate (3.43 g, 0.02 mol) in 130 mL of ethanol was then rapidly added to the chloro(triphenylphosphine) gold(I) solution. The mixture was stirred at 1000 rpm for 2 h in the absence of light. The obtained silver chloride precipitate was filtered out using sintered glass funnel porosity 3. The filtrate was then dried under reduced pressure using a rotary evaporator before washing with ethanol (3 x 50 mL). The remaining solid was dissolved in 30 mL of CH_2Cl_2 then filtered through a syringe filter. A 150 mL of ethanol was added prior to bubbling with the N_2 flow for 1 h. The white crystals obtained were collected by filtration. The product was washed with cold ethanol (3 x 10 mL) and cold diethyl ether (1 x 10 mL). The white crystals were dried in a vacuum desiccator in the absence of light. The product was transferred into a vial wrapped in tin foil and stored in a freezer (all manipulations were performed in the absence of light as much as possible). The yield of the pure $AuPPh_3NO_3$ product was *ca.* 2.97 g (0.006 mol, *ca.* 71 % by Au). ^{31}P-NMR ($CDCl_3$ over silver foil): singlet, δ 27.3 ppm relative to 85 % H_3PO_4, which agrees with those reported in the literature.[2]

S3.4 $Au_9(PPh_3)_8(NO_3)_3$

Au_9 was synthesized according to the protocol reported by Anderson *et al.* as mentioned in the experimental section.[2] Yield of the pure $AuPPh_3NO_3$ product was *ca.* 2.10 g, 60 ± 6 Au at%. ^{31}P-NMR (CD_2Cl_2, 25 °C); singlet, δ 57.5 ppm relative to H_3PO_4. ESI-MS

(CH_3OH/CH_3CN) exhibited the fragment ion of $[Au_9(PPh_3)_8]^{3+}$ at *m/z* of 1290. The identity of the pure cluster was also confirmed by PXRD.[2]

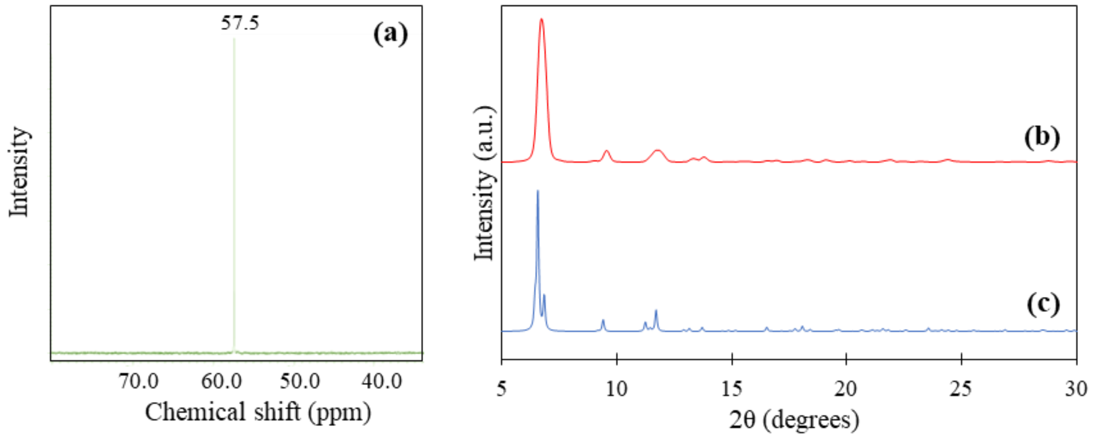

Figure S1. (a) ^{31}P-NMR (CD_2Cl_2, 25 °C) of $Au_9(PPh_3)_8(NO_3)_3$ nanoclusters, **(b)** experimental and **(c)** simulated PXRD pattern of $Au_9(PPh_3)_8(NO_3)_3$ nanoclusters.

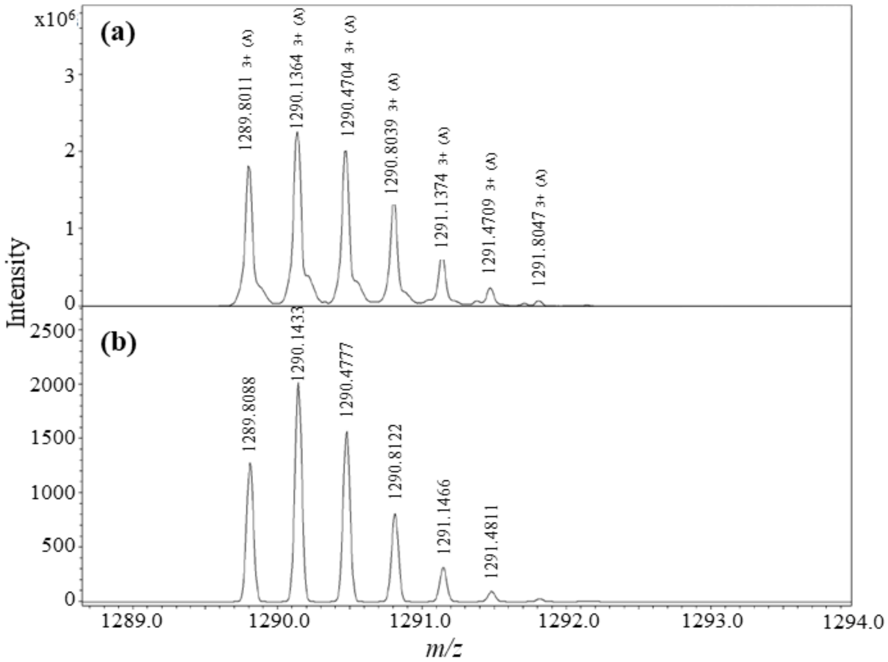

Figure S2. (a) experimental and **(b)** simulated spectra pattern for the $[Au_9(PPh_3)_8]^{3+}$ molecular ion peak at *m/z* of 1290.

S3.5 Au_9-MPTMS

The ligand exchange reaction of Au_9 with MPTMS ligands was performed following the procedure reported in the experimental section. The Au_9-MPTMS crude product was characterized by NMR, MS and UV-vis techniques. The ^{1}H-NMR (CD_3OD, 25 °C) of the crude Au_9-MPTMS product showed proton signals at (1) δ of 3.4, 2.8, 1.9 and 1.0 ppm relative to H_3PO_4 belonging to protons of MPTMS coordinated to Au_9, (2) δ of 3.6, 2.6, 1.7 and 0.8 ppm corresponding to protons of free MPTMS and (3) δ of 7.4 - 7.7 ppm belonging to the protons of free PPh_3 species. Success of the ligand exchange was suggested by the disappearance of the signal due to Au_9-bound phosphines at 58.46 ppm in ^{31}P-NMR and the absence of the $[Au_9(PPh_3)_8]^{3+}$ fragment ion (*m/z* of 1290) in MS. Partial decomposition

of the phosphine-protected Au_9 during the ligand exchange reaction was, however, evidenced by a small signal of $Au(PPh_3)Cl$ in ^{31}P-NMR δ of 33.0 - 33.9 ppm as well as the $[Au(PPh_3)_2]^+$ and $HPPh_3O^+$ fragment in MS at *m/z* of 721 and 279, respectively. Unfortunately, no clear evidence of the desired thiol stabilised Au_9 clusters was found in the MS. UV-vis spectrum (CH_3OH) of the $Au_9(PPh_3)_8(NO_3)_3$ cluster gave defined characteristic absorption peaks at λ_{max} of 314, 350, 382 and 445 nm. In contrast, those of the Au_9-MPTMS showed only broadened bands and shoulders at λ_{max} of 323, 369, 413, 447 and 705 nm, which could be attributed to the thiol-protected cluster product, as shown in **Figure 3 (b)**.

S3.6 Au_9-MPTMS@Na-LTA

The Au_9-MPTMS@Na-LTA prepared *via in situ* incorporation approach was demonstrated to be the most promising sample among all zeolites incorporating Au NCs made in this work. The reasons for this claim are that such material could be obtained in a great yield (96 ± 2 % based on SiO_2). Great consistency in phase crystallinity, degree of incorporated Au and absorption features are confirmed by PXRD, MP-AES analysis, and UV-vis DRS across eight batches synthesized, separately.

(a)

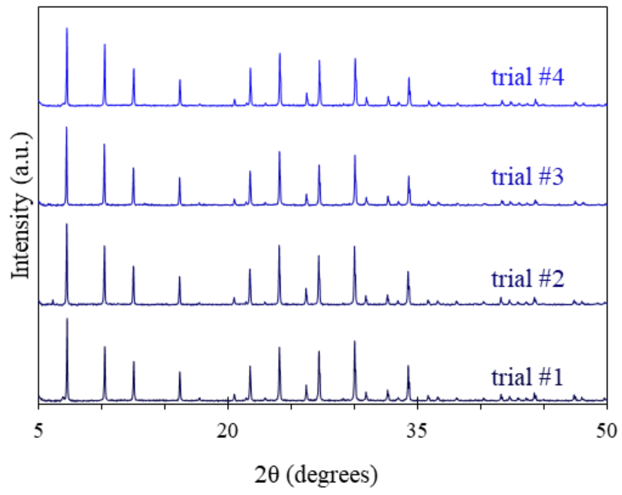

(b)

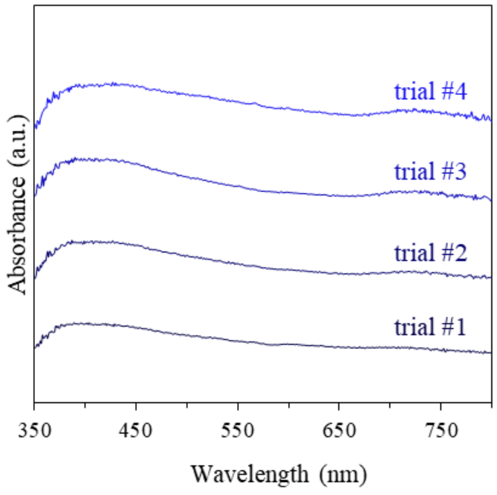

(c)

MP-AES results	
No.	Au content (wt%)
trial #1	1.08
trial #2	1.13
trial #3	1.10
trial #4	1.12
average	1.11 ± 0.02

Figure S3. (a) PXRD, **(b)** UV-DRS and **(c)** Au content of the Au_9-MPTMS@Na-LTA samples from four batches synthesized, separately.

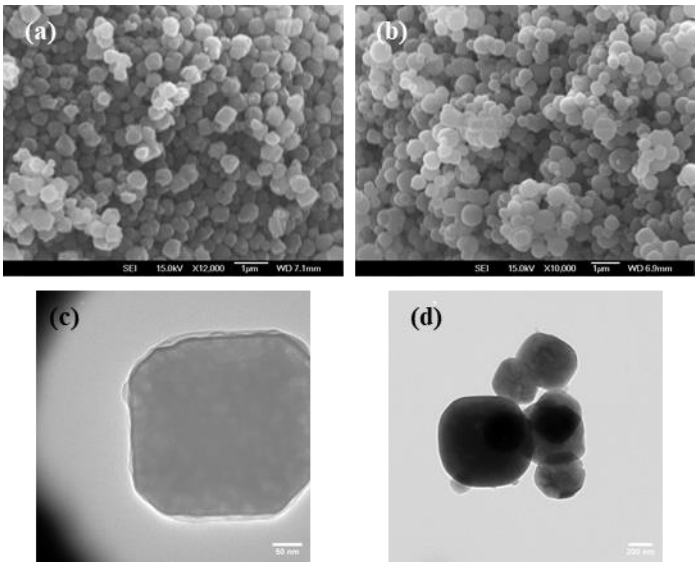

Figure S4. SEM images of **(a)** Na-LTA and **(b)** Au_9-MPTMS@Na-LTA (Scale bars are 1 μm) and **(c, d)** TEM images of Au_9-MPTMS@Na-LTA (Scale bars are 50 and 200 nm, respectively).

Thermogravimetric analysis (TGA) of the pure $Au_9(PPh_3)_8(NO_3)_3$ clusters, **Figure S5**, showed onset temperature for organic ligand removal of ~ 230 °C under N_2 flow. By continuing heating under the same condition, at 450 °C, the pure clusters lose ~ 56 wt% of the starting mass compared to the percentage of organic ligands calculated from empirical formula (56.3 wt%). Unlike TGA of pristine Au NCs, zeolite incorporated Au NCs showed no sharp weight loss across the monitoring temperature. There was no distinct difference in the TGA curves of Au_9-MPTMS@Na-LTA compared to that of pure Na-LTA or MPTMS@Na-LTA. This was due to sensitivity limits of TGA since the loss of MPTMS in the samples could only cause less than 1 wt% loss. In contrast, ~ 25 wt% loss of all zeolite-based samples at lower than 400 °C was attributed to trapped solvent and/or moisture in the zeolite voids, possibly, together with MPTMS ligands. Thus, the decomposition temperature of individual species in Au_9-MPTMS@Na-LTA cannot be identified.

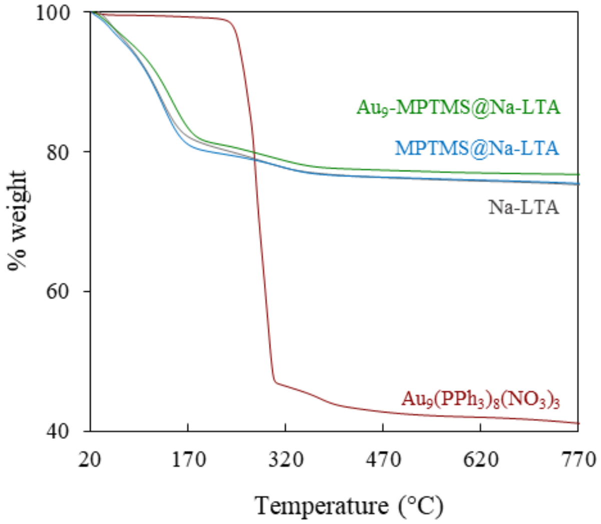

Figure S5. TGA curves of Au_9-MPTMS@Na-LTA comparing with that of MPTMS@Na-LTA, Na-LTA and pure $Au_9(PPh_3)_8(NO_3)_3$ nanoclusters.

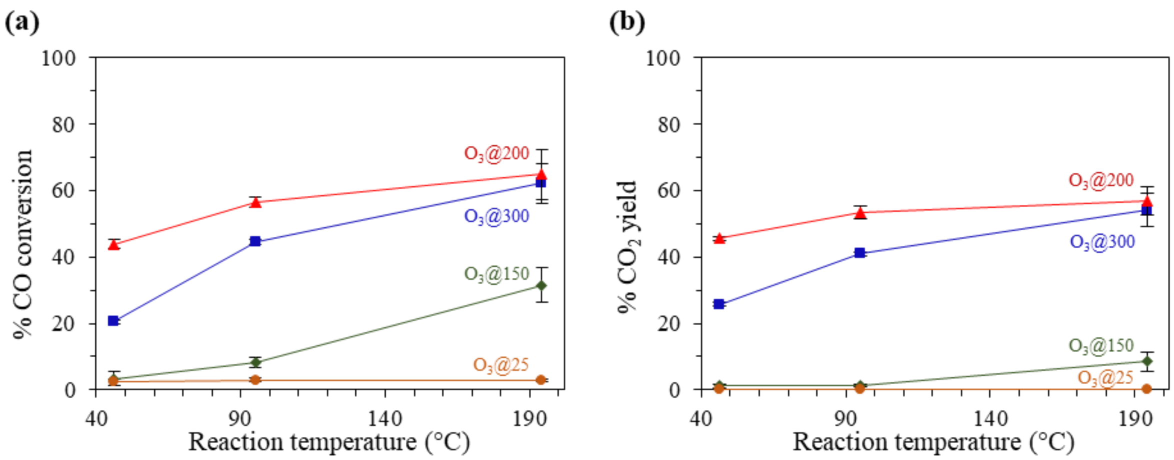

Figure S6. **(a)** % CO conversion and **(b)** % CO_2 yield in CO oxidation catalysed by O_3-treated Au_9-MPTMS@Na-LTA (0.75 ± 0.01 wt% Au) - ozonolysis temperature of 25, 150, 200 and 300 °C. Reaction conditions: GHSV of 3,000 mL g^{-1} h^{-1}, 200 mg_{cat}, 50 - 200 ºC. The error bars represent the standard deviation of multiple replicates of the same experiment (n ≥ 3).

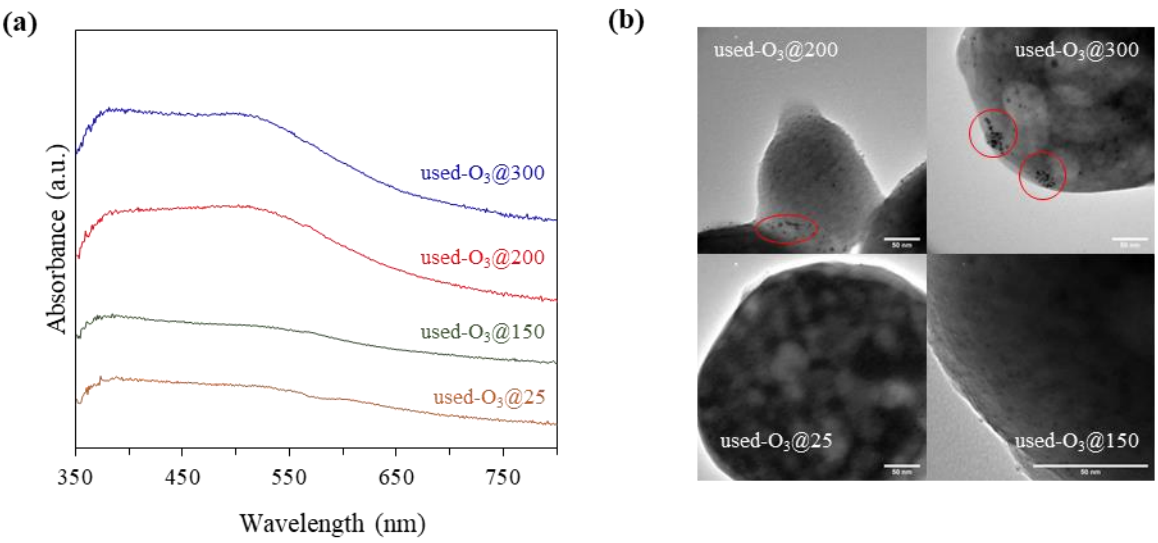

Figure S7. **(a)** UV-vis DRS and **(b)** TEM images of post-reaction samples of O_3-treated Au_9-MPTMS@Na-LTA samples - ozonolysis temperature of 25, 150, 200, and 300 °C. Reaction conditions: T_{max} of 200 °C, a total reaction time of 12 h. Scale bars are 50 nm.

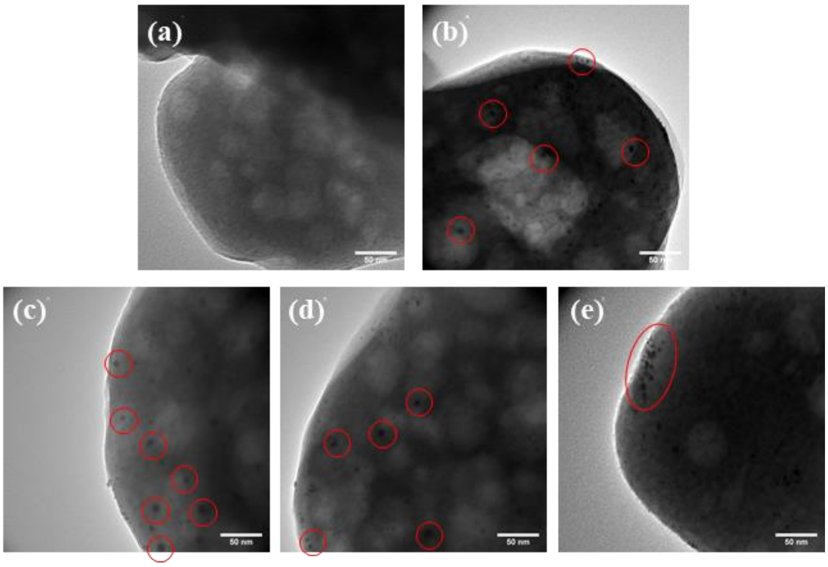

Figure S8. TEM images of **(a)** O_3@200-treated Au_9-MPTMS@Na-LTA and the post-reaction samples tested at **(b)** 30, **(c)** 50, **(d)** 100 and **(e)** 200 ºC. (Reaction conditions: temperature of 30, 50, 100 and 200 °C, a reaction time of 2 h) Au NPs are highlighted in red circles.
Scale bars are 50 nm.

S3.7 Au_9-MPTMS@Na-FAU

Au_9-MPTMS@Na-FAU was fabricated using the *in situ* incorporation approach, analogues to the Au_9-MPTMS@Na-LTA. Both zeolites were hydrothermally treated at a similar crystallization condition (see in **Experimental**). However, an average % yield of Au_9-MPTMS@Na-FAU samples (30 ± 1 % based on SiO_2 or 65 ± 4 % based on Al_2O_3) was significantly lower than that of Au_9-MPTMS@Na-LTA (96 ± 2 % based on SiO_2) which was also lower than those reported in the literature (~ 98 % based on Al_2O_3).[3] The higher Na-FAU yield could be achieved with the assistance of an organic template (tetramethylammonium cation) or by using the seed gel approach with a longer crystallization period. However, it might be important to avoid significant differences in synthesis parameters that could affect zeolite crystallite size. Hence, the synthesis of Au_9-MPTMS@Na-FAU was attempted under synthesis conditions as close as possible to those used for Au_9-MPTMS@Na-LTA one.

Structural evidence of the FAU zeolite phase and their purity were verified by PXRD analysis (**Figure S9)**. Even with the lower yield, the crystallinity of Au_9-MPTMS@Na-FAU was comparable to that of the commercial one as suggested by a similar PXRD peaks intensity. An absence of visible amorphous phase in SEM images, **Figure S10** further confirmed the purity of Na-FAU crystalline samples. The presence of Au_9-MPTMS did not show a visible effect on the FAU crystallite shape or size. It was also barely affected the crystallinity of Na-FAU zeolite as confirmed by unchanged PXRD peak intensity, similar to those found in the Na-LTA case. Crystallite sizes of Na-FAU-based (**Figure S10**) and Na-LTA-based (**Figure S4 (a, b)**) samples was found to be in a similar range of 200 - 700 nm. Both zeolites were hydrothermally synthesized under a similar condition where an absence of structure-directing agents was responsible for the large crystallite size variation.

The Au_9-MPTMS@Na-FAU was a uniform in color pale cream fine powder. However, the sample showed a very low Au content of 0.40 ± 0.01 wt% when 1.0 wt% Au was added to the synthesis gel. To achieve Au_9-MPTMS@Na-FAU with 0.96 ± 0.05 wt% Au, 2.4 wt% Au was introduced into the synthesis gel. UV-vis DRS of the Au_9-MPTMS@Na-FAU shown in **Figure 8** suggested that a higher density of Au NCs was located at the zeolite crystallite's outer surface as discussed in **Results and discussion** section.

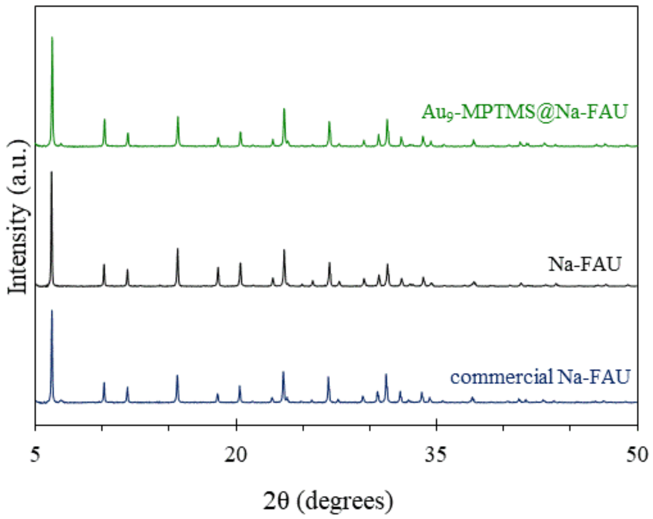

Figure S9. PXRD of Na-FAU zeolite-based samples synthesized with and without the addition of Au_9-MPTMS comparing with that of commercial Na-FAU (Linde Type Y).

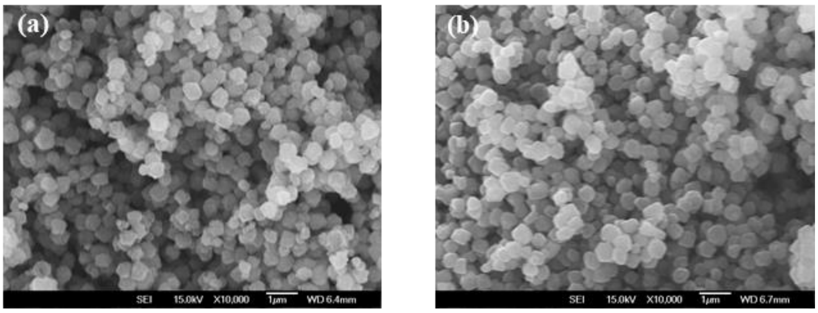

Figure S10. SEM images of **(a)** Na-FAU and **(b)** Au_9-MPTMS@Na-FAU. Scale bars are 1 μm.

S3.8 AuNPs-MPTMS@Na-LTA

The AuNPs-MPTMS@Na-LTA was prepared following the procedure reported by Iglesia *et al.* with slight modifications.[4] A colloidal SiO_2 and $NaAlO_2$ were added to an aqueous solution of $HAuCl_4$ and MPTMS. The Au-containing aluminosilicate gel was aged at ambient temperature for 2 h before hydrothermal treatment at 100 ºC under the autogenous pressure with stirring (750 rpm) for 14 h. The AuNPs-MPTMS@Na-LTA product was collected by centrifugation, washed with Mill-Q water once, and soaked in methanol overnight before drying at 100 ºC. The AuNPs-MPTMS@Na-LTA was obtained with 98 ± 2 % yield based on SiO_2, comparable to the yield reported in the literature and that of the Au_9-MPTMS@Na-LTA. PXRD analysis verified the LTA zeolite phase and crystallinity. As shown in **Figure S11**, the AuNPs-MPTMS@Na-LTA and Au_9-MPTMS@Na-LTA was found to have a similar crystallinity.

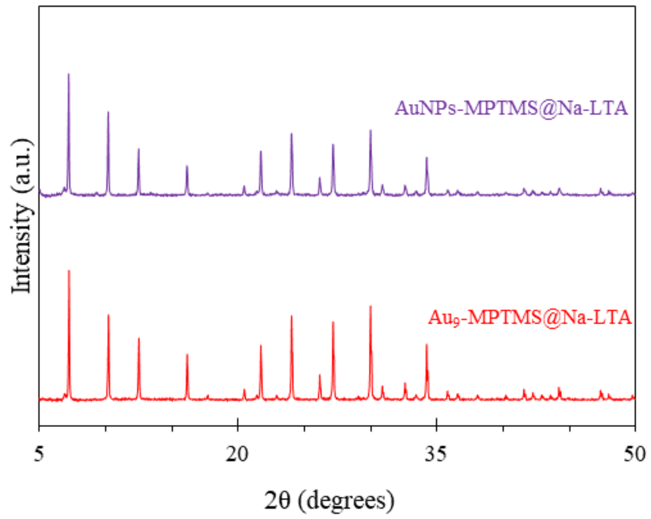

Figure S11. PXRD of AuNPs-MPTMS@Na-LTA and Au_9-MPTMS@Na-LTA with Au content of 1.16 ± 0.02 wt% Au and 1.11 ± 0.02 wt% Au, respectively.

S3.9 Au-free Na-LTA zeolite

Na-LTA zeolite was synthesized according to the procedure reported by Wu *et al.*[23] Briefly, NaOH (4.8 g, 0.12 mol) and $NaAlO_2$ (6.0 g, 0.07 mol) were dissolved in 62 mL of Milli-Q water. Fumed silica (3.2 g, 0.05 mol) was then added to aluminate solution with stirring, resulting in a homogeneous cream-gel with the composition of 2.6 Na_2O: 1.0 Al_2O_3:1.5 SiO_2: 93.6 H_2O. The zeolite synthesis gel was further stirred at 60 °C for 4 h. The aged gel was transferred into autoclaves and crystallized at 100 °C for 16 h. The solid product was collected by centrifugation, washed with Milli-Q water and further soaked in methanol overnight. The Na-LTA was dried overnight under vacuum and further dried at 100 °C for 12 h in ambient air. The yield of the Na-LTA product was *ca*. 8.12 g (81 ± 3 % based on SiO_2).

S3.10 imp-Au_9-MPTMS@Na-LTA

For comparison, post-impregnated samples were prepared by solution-based deposition. The pre-made Na-LTA were suspended in ligand-protected Au_9 clusters solutions. Typically, Na-LTA (9.72 g, 0.56 mol) was added to a 50 mL-methanolic solution of Au_9-MPTMS aiming for 1.0 wt% Au loading final samples. The suspension was stirred at ambient temperature for 20 h. The sample was then collected by centrifugation at 12,000 rpm for 5 min. Decanting mother liquor, final solid product, denoted as 'imp-Au_9-MPTMS@Na-LTA' were obtained. The sample was collected and dried overnight at ambient temperature under vacuum. The sample was transferred into a vial, sealed, and stored in a freezer.

S4. Catalytic CO oxidation

The catalytic CO oxidation was performed in a continuous-flow fixed-bed reactor connected to an online gas chromatograph (GC).

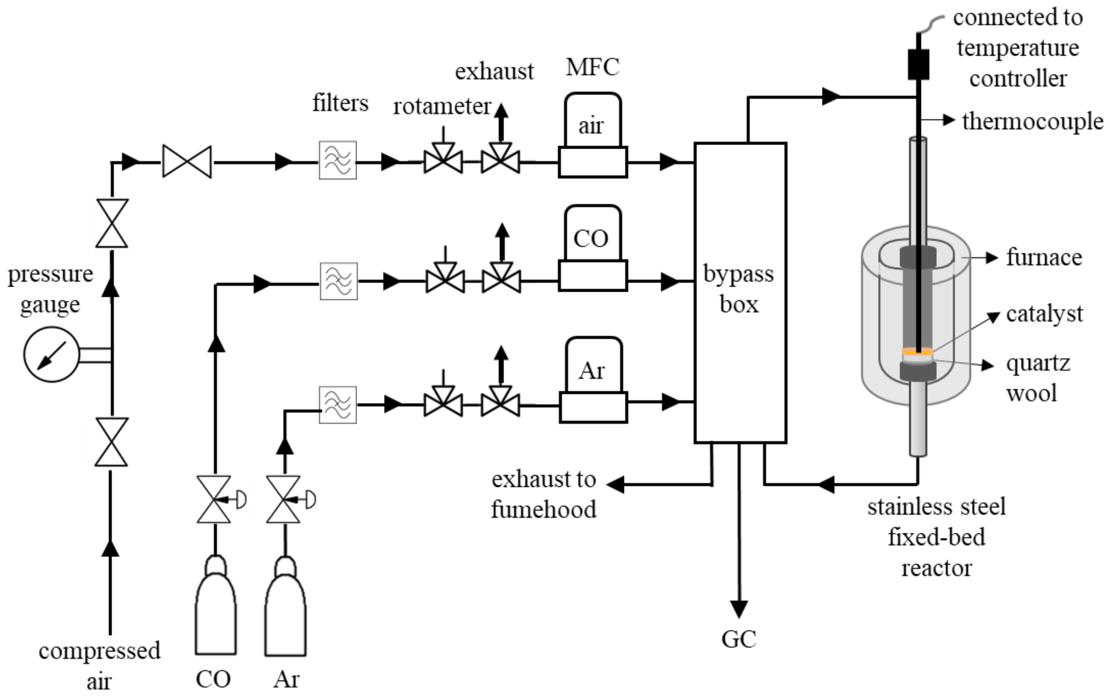

Figure S12. Process flow diagram of the fixed-bed reactor used for CO oxidation.

The mixture of gases passed through the catalyst bed was collected and analyzed by the online GC (SRI Multiple Gas Analyzer, MG #3) equipped with a molecular sieve 13X (washed) 80/100 packed column (6' x 1/8" x 0.085 ss) and a Hayesep D 80/100 packed column (6' x 1/8" x 0.085 ss) using Argon as a carrier gas. FID equipped with a methanizer and TCD detectors were used in this system to detect CO and CO_2 gases in low concentrations. The GC parameters and column heating program are shown in **Table S1**.

Table S1. GC setting for analysis of CO oxidation reaction mixtures.

GC setting for CO oxidation	
FID temperature (°C)	300
TCD temperature (°C)	150
Methanizer temperature (°C)	300
FID gain	High gain (filtered)
TCD gain	Low gain
Injection volume (mL)	1.0
Column flow (mL/min)	20, Ar as carrier gas
GC column heating program	80 °C, 14.5 min

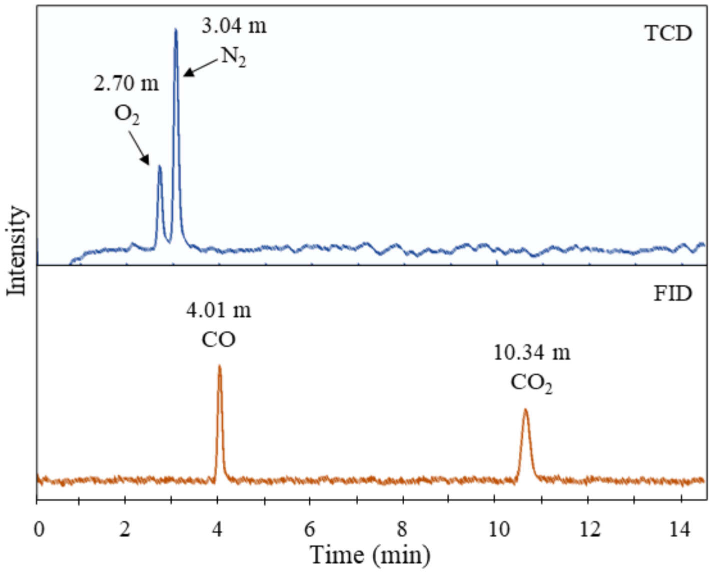

Figure S13. Typical chromatograms of CO oxidation reaction obtained using FID and TCD detectors.

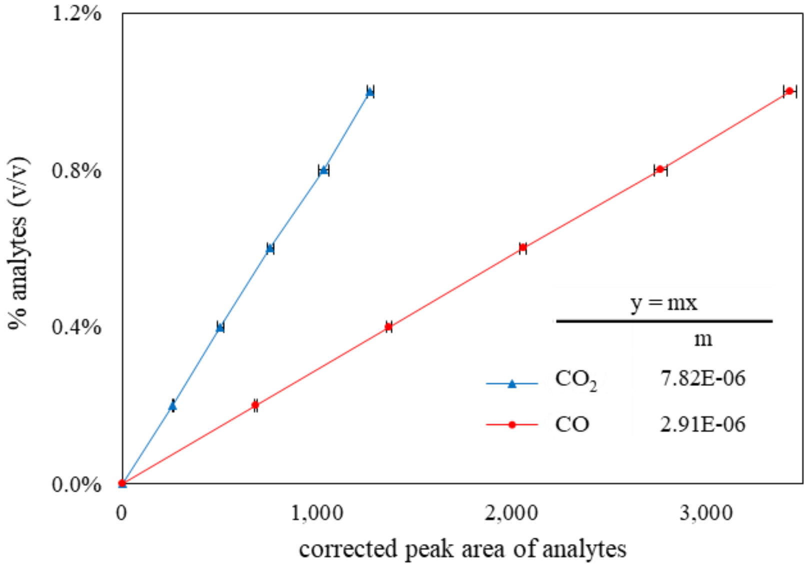

Figure S14. Calibration plots for CO oxidation reaction.

Conversion of CO and yield of CO_2 was calculated using following equations:

$$unconverted\ CO\ (\%v/v) = response\ factor\ \times\ peak\ area\ _{CO}$$

$$produced\ CO_2\ (\%v/v) = response\ factor\ \times\ peak\ area\ _{CO_2}$$

$$\%\ CO\ conversion = \frac{initial\ CO\ (\%v/v) - \ unconverted\ CO\ (\%v/v)}{initial\ CO\ (\%v/v)} \times 100$$

$$\%\ CO_2\ yiled = \frac{produced\ CO_2\ (\%v/v)}{initial\ CO\ (\%v/v)} \times\ 100$$

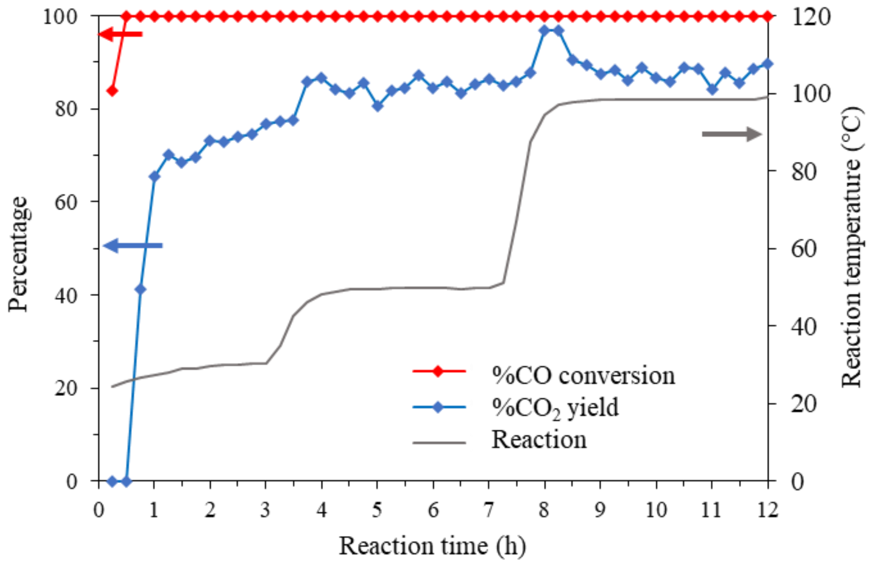

Figure S15. % CO conversion and % CO_2 yield in CO oxidation catalyzed by O3@200-inst-Au_9-MPTMS@Na-LTA (1.11 ± 0.02 wt% Au). Reaction conditions: GHSV of 3,000-mL g^{-1} h^{-1}, a total Au loading of 2.2 mg, 30 - 100 ºC.

References

1. Glemser, O.; Sauer, H. Copper, silver, gold. In *Handbook of Preparative Inorganic Chemistry*, 2nd ed.; Brauer, G., Ed. Academic Press Inc.: London, UK, 1963; Volume 1.
2. Anderson, D. P.; Alvino, J. F.; Gentleman, A.; Qahtani, H. A.; Thomsen, L.; Polson, M. I. J.; Metha, G. F.; Golovko, V. B.; Andersson, G. G. Chemically-synthesised, atomically-precise gold clusters deposited and activated on titania. *Phys. Chem. Chem. Phys.* **2013,** *15*, 3917-3929.
3. Ginter, D. Chapter 46 - FAU Linde Type Y Si(71), Al(29). In *Verified Syntheses of Zeolitic Materials*; Robson, H.; Lillerud, K. P., Eds.; Elsevier: Amsterdam, The Netherland, 2001; pp 156-158.
4. Otto, T.; Zones, S. I.; Iglesia, E. Challenges and strategies in the encapsulation and stabilization of monodisperse Au clusters within zeolites. *J. Catal.* **2016,** *339*, 195-208.
